# Mechanism of differential Zika and dengue virus neutralization by a public antibody lineage targeting the DIII lateral ridge

**DOI:** 10.1084/jem.20191792

**Published:** 2019-11-22

**Authors:** Haiyan Zhao, Lily Xu, Robin Bombardi, Rachel Nargi, Zengqin Deng, John M. Errico, Christopher A. Nelson, Kimberly A. Dowd, Theodore C. Pierson, James E. Crowe, Michael S. Diamond, Daved H. Fremont

**Affiliations:** 1 Department of Pathology & Immunology, Washington University School of Medicine, Saint Louis, MO; 2 The Vanderbilt Vaccine Center, Vanderbilt University Medical Center, Nashville, TN; 3 Department of Cell Biology & Physiology, Washington University School of Medicine, Saint Louis, MO; 4 Viral Pathogenesis Section, National Institute of Allergy and Infectious Diseases, National Institutes of Health, Bethesda, MD; 5 Department of Molecular Microbiology, Washington University School of Medicine, Saint Louis, MO; 6 Department of Medicine, Washington University School of Medicine, Saint Louis, MO; 7 Department of Biochemistry & Molecular Biophysics, Washington University School of Medicine, Saint Louis, MO; 8 Department of Pediatrics, Vanderbilt University Medical Center, Nashville, TN; 9 Department of Pathology, Microbiology and Immunology, Vanderbilt University Medical Center, Nashville, TN

## Abstract

We previously generated a panel of human monoclonal antibodies (mAbs) against Zika virus (ZIKV) and identified one, ZIKV-116, that shares germline usage with mAbs identified in multiple donors. Here we show that ZIKV-116 interferes with ZIKV infection at a post-cellular attachment step by blocking viral fusion with host membranes. ZIKV-116 recognizes the lateral ridge of envelope protein domain III, with one critical residue varying between the Asian and African strains responsible for differential binding affinity and neutralization potency (E^393^D). ZIKV-116 also binds to and cross-neutralizes some dengue virus serotype 1 (DENV1) strains, with genotype-dependent inhibition explained by variation in a domain II residue (R^204^K) that potentially modulates exposure of the distally located, partially cryptic epitope. The V-J reverted germline configuration of ZIKV-116 preferentially binds to and neutralizes an Asian ZIKV strain, suggesting that this epitope may optimally induce related B cell clonotypes. Overall, these studies provide a structural and molecular mechanism for a cross-reactive mAb that uniquely neutralizes ZIKV and DENV1.

## Abstract (precis)

Evaluation of the human antibody response to Zika virus has identified common germline-derived mAbs capable of cross flavivirus neutralization. Zhao et al. provide a detailed mechanistic understanding of how flavivirus infections are prevented in a strain-specific manner by a representative mAb.

## Graphical Abstract

**Figure:**
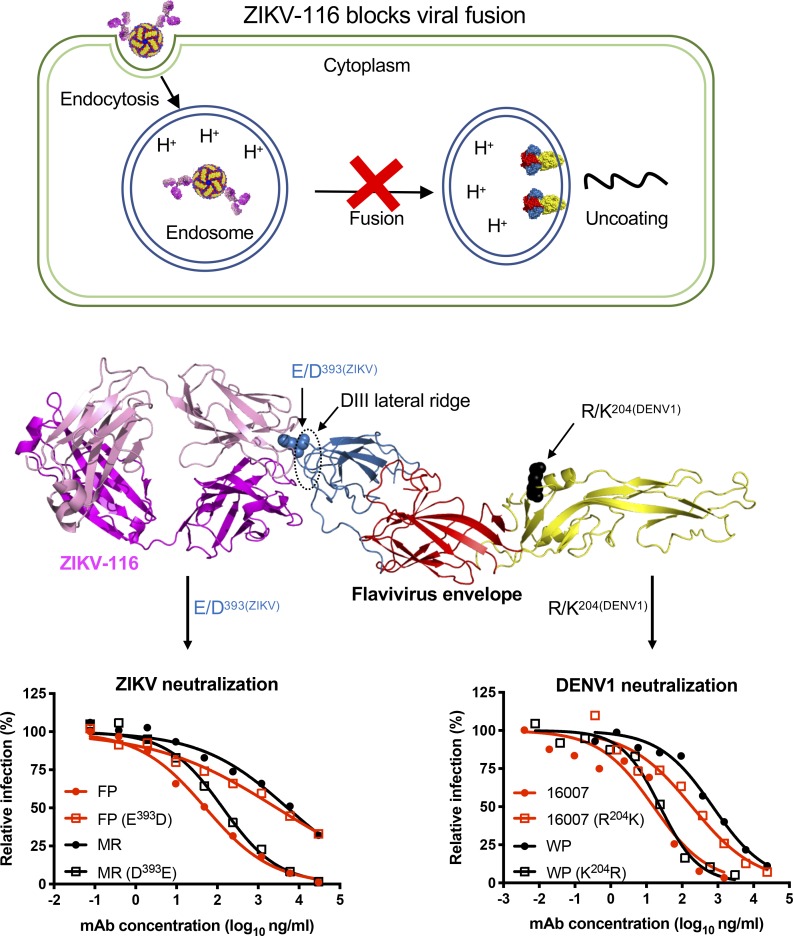

## Introduction

Zika virus (ZIKV) typically causes a self-limiting febrile illness, with most infected individuals exhibiting minimal or no symptoms (Duffy et al., 2009). However, ZIKV infection can result in severe neurological disease (Mlakar et al., 2016), including neurodevelopmental defects in infants after congenital infection (Moore et al., 2017; de Paula Freitas et al., 2016). Dengue virus (DENV) is genetically related to ZIKV, infects nearly 400 million people annually, and causes variable clinical disease ranging from a mild to severe febrile illness and life-threatening dengue shock syndrome (Bhatt et al., 2013). Since its introduction and spread in the Western hemisphere in 2015–2016, ZIKV has emerged as a significant global health concern.

Both ZIKV and DENV are principally transmitted by mosquitoes (Cao-Lormeau et al., 2016) and belong to the *Flavivirus* genus of the Flaviviridae family of single-stranded positive-sense RNA viruses, which also include West Nile (WNV), Japanese encephalitis (JEV), yellow fever, and the tick-borne encephalitis viruses (Lazear and Diamond, 2016). Flavivirus genomes encode a single polyprotein that is cleaved by viral and cellular proteases into three structural proteins (capsid protein, precursor membrane protein, and envelope [E] protein) and seven nonstructural proteins. Cryo-electron microscopy (cryo-EM) models of mature flaviviruses show 90 anti-parallel E protein dimers lying flat against the virion surface with T = 3 quasi-icosahedral symmetry (Zhang et al., 2013; Kostyuchenko et al., 2016; Qiu et al., 2018). E protein is the primary target of neutralizing antibodies and is composed of three ectodomains: domain I (DI), which links DII and DIII together; DII, which contains a fusion loop that mediates viral fusion with host endosomes; and DIII, which adopts an Ig-like fold that undergoes a substantial repositioning during viral fusion (Rey et al., 1995; Dai et al., 2016; Modis et al., 2004). Antibodies against flaviviruses map to epitopes in all three domains, and those against DIII are among the most potent at neutralizing infection (Nybakken et al., 2005; Robbiani et al., 2017; Zhao et al., 2016; Shrestha et al., 2010; Sukupolvi-Petty et al., 2010).

While the affinity of antibody binding governs the proportion of epitopes occupied under steady state conditions (Robinson et al., 2015), it does not always correlate with flavivirus neutralization. Another factor that influences antibody neutralization is the valency of virion engagement, where potent neutralization can be obtained for a bivalent binding antibody even in the setting of relatively weak monovalent affinity (Edeling et al., 2014). A third important factor is epitope accessibility, which is influenced by virion maturation as well as the capacity for dynamic motion and affects the stoichiometry of antibody binding and efficiency of neutralization (Pierson et al., 2007; Pierson and Diamond, 2012).

Germline selection and affinity maturation of broadly neutralizing mAbs have been studied extensively for HIV and influenza virus and have allowed for the development of novel vaccine strategies (Pappas et al., 2014; Liao et al., 2013; Duan et al., 2018). Germline precursors generally show weak or undetectable affinity for target immunogens; thus, vaccine antigens may need to be engineered to induce neutralizing antibodies. For flaviviruses, most cross-reactive mAbs against the E protein target the highly conserved fusion loop in DII. The accessibility of the fusion loop is dependent on the maturation state of the virus, with limited exposure on mature virions, and most fusion loop–directed mAbs exhibit weak neutralization potency (Zhao et al., 2016; Cherrier et al., 2009; Rey et al., 2018). Another group of cross-reactive mAbs has also been identified from DENV-infected donors that bind a quaternary E-dimer epitope and can neutralize both DENV and ZIKV infection efficiently (Dejnirattisai et al., 2015; Fernandez et al., 2017). These E-dimer epitope mAbs have shown significant potency against ZIKV both prophylactically and therapeutically in murine models of infection (Fernandez et al., 2017).

We and others have reported mAbs from multiple human donors that all use the V_H_3–23 heavy chain and V_K_1–5 light chain and have the unique capacity to neutralize both ZIKV and DENV1 with varying potencies (Sapparapu et al., 2016; Robbiani et al., 2017; Magnani et al., 2017; Niu et al., 2019). Several of these public lineage mAbs have demonstrated protection in animal models of ZIKV challenge. Herein, we combine virological and biophysical assays to define the mechanistic basis for the neutralizing activity of ZIKV-116, a mAb derived from a donor infected by ZIKV in 2015 in Brazil. Functional studies in cell culture show that ZIKV-116 primarily inhibits infection at a post-cellular attachment step by blocking viral fusion with host membranes. X-ray crystallographic studies reveal that ZIKV-116 recognizes a DIII lateral ridge (LR) epitope, with one residue distinguishing binding and consequent neutralization of Asian versus African ZIKV strains. ZIKV-116 also potently neutralizes infection of DENV1, with genotype-specific neutralization best explained by differential epitope exposure. Lastly, we constructed the inferred V-J germline sequence of ZIKV-116 and demonstrated that it binds and neutralizes diverse ZIKV and DENV1 strains, albeit with lower potency than the affinity-matured clone.

## Results

### ZIKV-116 primarily inhibits infection at a post-attachment step

**Figure 1.**
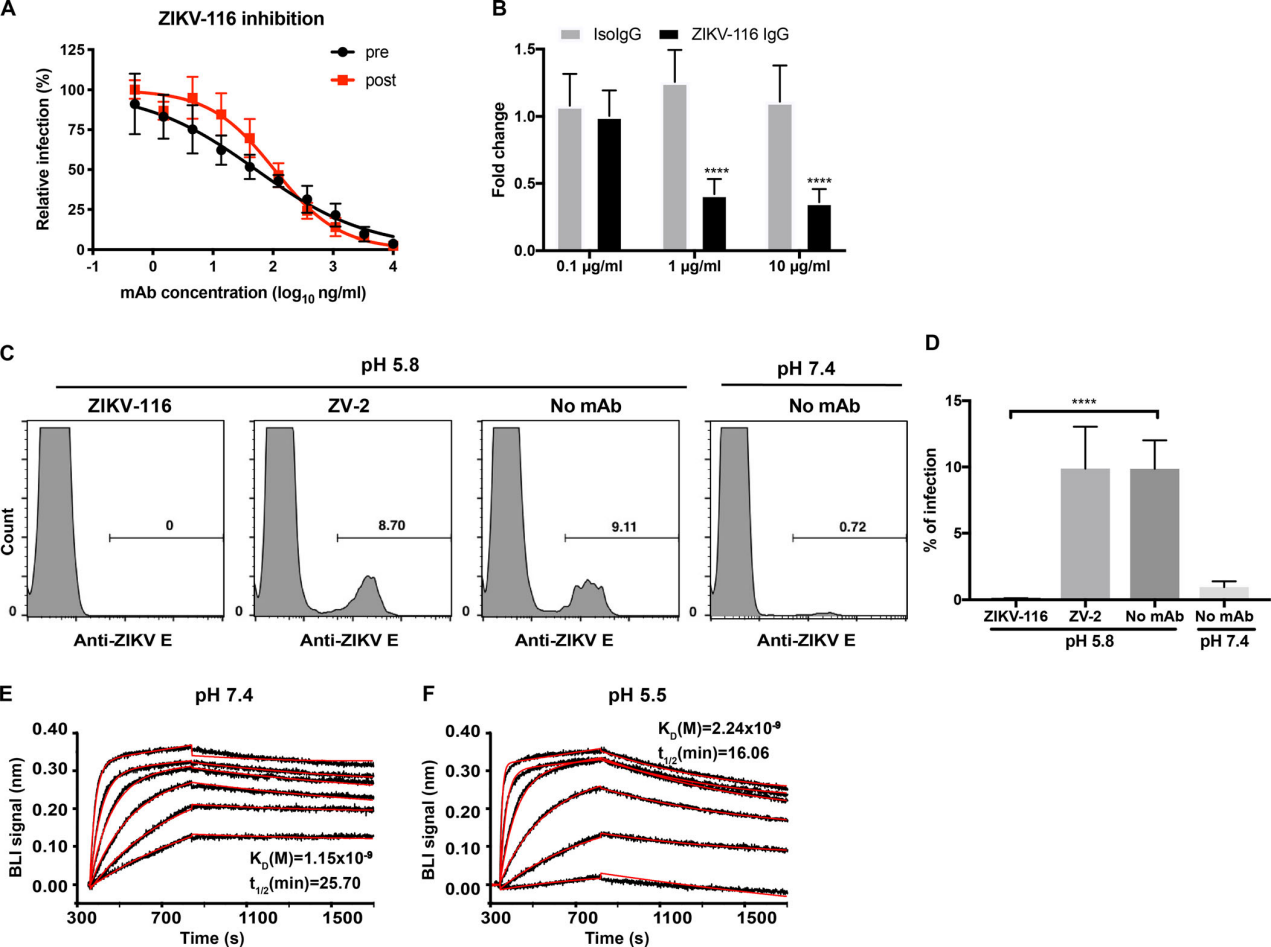
**The mechanistic basis of ZIKV-116 neutralization**. **(A)** Pre-/post-attachment inhibition assay. Serial dilutions of mAb were added to the Vero cells before (pre, black line) or after (post, red line) virus adsorption to the cells, and infection was determined by FFA. Wells containing mAb were compared with wells containing no mAb to determine the relative infection. **(B)** Attachment blockade assay. ZIKV (H/PF/2013) was incubated with ZIKV-116 or isotype control (human IgG1) for 1 h, followed by addition to pre-chilled Vero cells for 1 h. Bound ZIKV RNA was measured by qRT-PCR. GAPDH was used for an internal control, and viral RNA fold change was compared with control cells incubated in the absence of mAb. Data are the mean of three independent experiments performed in duplicate or quadruplicate. Error bars indicate SD. **(C and D)** Fusion blockade assay. Pre-chilled Vero cells were incubated with ZIKV (multiplicity of infection of 80) for 1 h on ice. Subsequently, 100 µg/ml of ZIKV-116 IgG, control ZV-2 IgG (nonneutralizing anti-ZIKV mAb), or medium was added for 1 h on ice, and the pH was shifted to 5.8 to trigger virus fusion with plasma membrane. After pH normalization, cells were incubated at 37°C for 24 h, and the number of infected cells was determined by flow cytometry. 25 mM NH_4_Cl was included in the medium throughout the procedure. Representative flow cytometric histogram (C). The data averaged from two independent experiments are shown as means with SD (D). **(E and F)** Representative BLI profile of ZIKV-116 to recombinant H/PF/2013 DIII at neutral (pH 7.4) and acidic pH (pH 5.5). The experimental curves (black lines) were fitted using a 1:1 Langmuir binding analysis (red lines), and the results are representative of three independent experiments. Statistical significance was determined by two-tailed *t* test (D) or two-way ANOVA with Sidak's post-test (B). ****, P < 0.0001.

To investigate how ZIKV-116 inhibited virus infection, we performed pre- and post-attachment neutralization assays with a French Polynesian ZIKV strain (H/PF/2013). As anticipated, prebinding with ZIKV-116 efficiently limited ZIKV infection (Fig. 1 A). ZIKV-116 similarly inhibited ZIKV infection after virus was bound to the target cell surface (half maximal inhibition concentration [IC_50_] of 52 and 105 ng/ml for pre- and post-attachment neutralization, respectively). To corroborate these studies, we evaluated whether ZIKV-116 could inhibit cellular attachment of ZIKV. Different concentrations of ZIKV-116 or an isotype control mAb were preincubated with ZIKV and then added to Vero cells. After extensive washing, cell-associated viral RNA was measured by quantitative real-time PCR (qRT-PCR). ZIKV-116 did not block virus attachment relative to isotype control near the IC_50_ of 0.1 µg/ml (Fig. 1 B). However, a modest inhibition of attachment (∼60%) was observed at concentrations ∼10- to 100-fold higher than the IC_50_ value, suggesting that attachment blockade is not the dominant inhibitory mechanism of ZIKV-116.

### ZIKV-116 can block viral fusion with host membranes

We next examined whether ZIKV-116 could block viral fusion. Normally, flaviviruses enter cells via receptor-mediated internalization, and pH-triggered fusion occurs with endosomal membranes (Modis et al., 2004). However, under low-pH conditions, cell-associated virus can be induced to fuse at the plasma membrane, a process termed “fusion from without” (Edwards and Brown, 1986; Liao and Kielian, 2005; Thompson et al., 2009; Fernandez et al., 2018). ZIKV was preadsorbed to Vero cells on ice and subsequently treated with ZIKV-116 or control ZV-2, a ZIKV-specific nonneutralizing mAb (Zhao et al., 2016). Virus-plasma membrane fusion was triggered by a brief exposure to low pH, and infection was monitored at 24 h after initial treatment. In all steps, 25 mM NH_4_Cl was added to prevent ZIKV entry and infection via endosomal fusion (Helenius et al., 1982). As expected, under neutral pH conditions, ZIKV infection was rarely detected. In comparison, exposure of cell-adsorbed ZIKV to pH 5.8 resulted in a 10-fold increase in infection. ZIKV-116 completely blocked this pH-triggered infection, whereas mAb ZV-2 did not (Fig. 1, C and D).

To block fusion in cells, ZIKV-116 must retain binding at the acidic pH of the endosome. To evaluate this question, we performed biolayer interferometry (BLI) with ZIKV-116 and its ligand ZIKV DIII at pH values corresponding to the extracellular and endosomal milieu. ZIKV-116 was immobilized and dipped into solutions of recombinant ZIKV DIII (H/PF/2013) ranging from 3.125 nM to 100 nM concentration. At pH 7.4, DIII bound to ZIKV-116 with a kinetically derived binding affinity (K_D_) of 1.15 nM and *t*_1/2_ of 25.70 min. The complex was only slightly less stable at pH 5.5, with a K_D_ of 2.24 nM and *t*_1/2_ of 16.06 min (Fig. 1, E and F). These results suggest that ZIKV-116 engagement of virions should endure within the acidic environment of endosomes such that viral fusion can be blocked.

### X-ray crystal structure of ZIKV-116 in complex with ZIKV DIII

**Figure 2.**
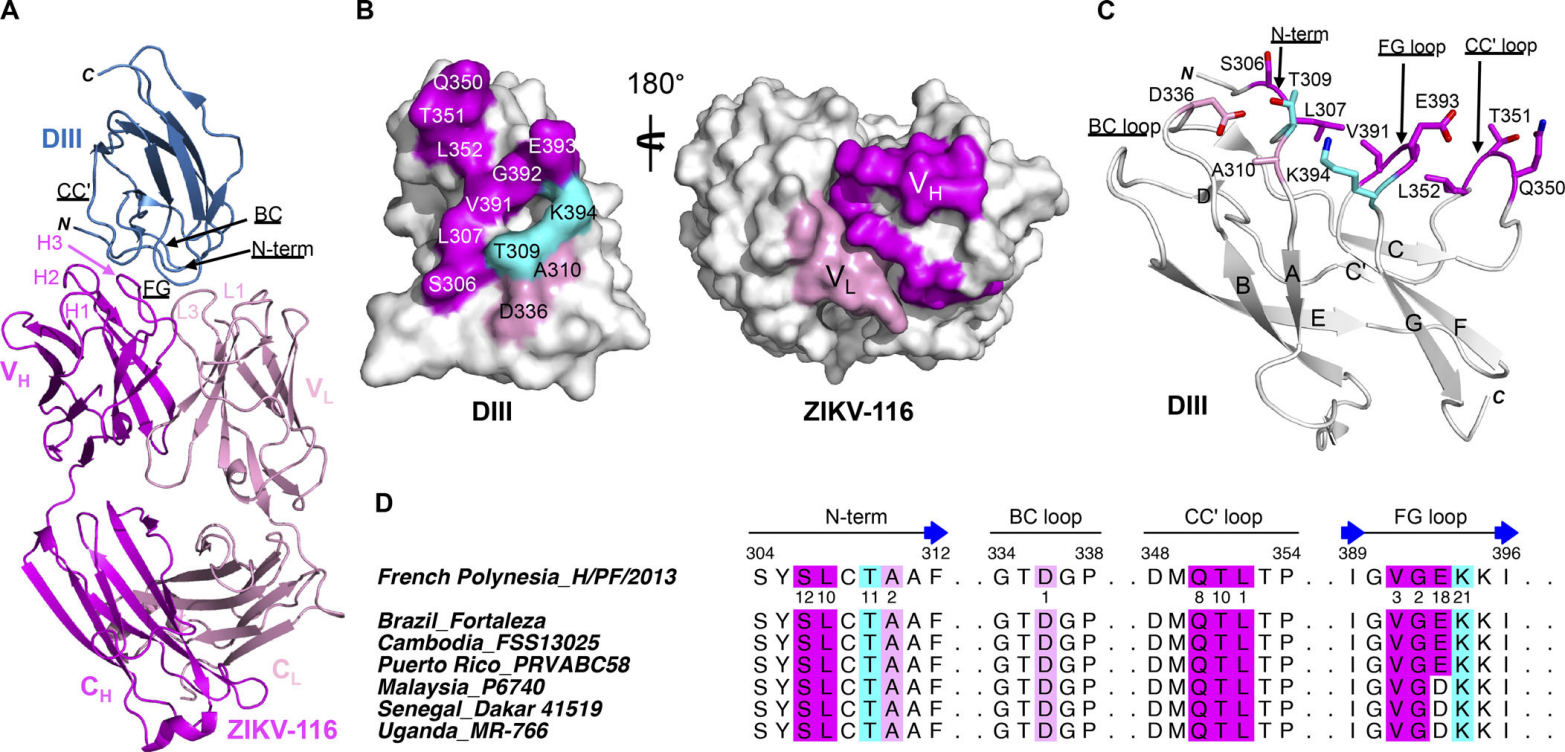
**ZIKV-116 in complex with ZIKV H/PF/2013 DIII.** **(A)** Ribbon diagram of the crystal structure, with DIII colored in sky blue, heavy chain in magenta, and light chain in pink. **(B)** Surface model of ZIKV DIII (left) and ZIKV-116 Fab (right) showing the interface of the complex. DIII contacts are displayed by heavy chain (magenta), light chain (pink), or both chains (cyan). Fab contacts are highlighted in magenta for heavy chain and pink for light chain. **(C)** The structural epitope on ZIKV DIII contacted by the ZIKV-116 is shown as sticks and colored as in B. **(D)** ZIKV-116 contacts on the four segments of DIII amino acid sequence alignment of different ZIKV strains. The DIII residues that make van der Waals contact distance <3.90 Å are colored as in B, and the numbers below the H/PF/2013 DIII represent the total number of contacts for each residue. Asian genotype: French Polynesia_H/PF/2013, Brazil_Fortaleza, Cambodia_FSS13025, Puerto Rico_PRVABC58, Malaysia_P6740; African genotype: Senegal_Dakar 41519, Uganda_MR-766. V_H_, heavy chain variable domain; V_L_, light chain variable domain.

**Figure S1.**
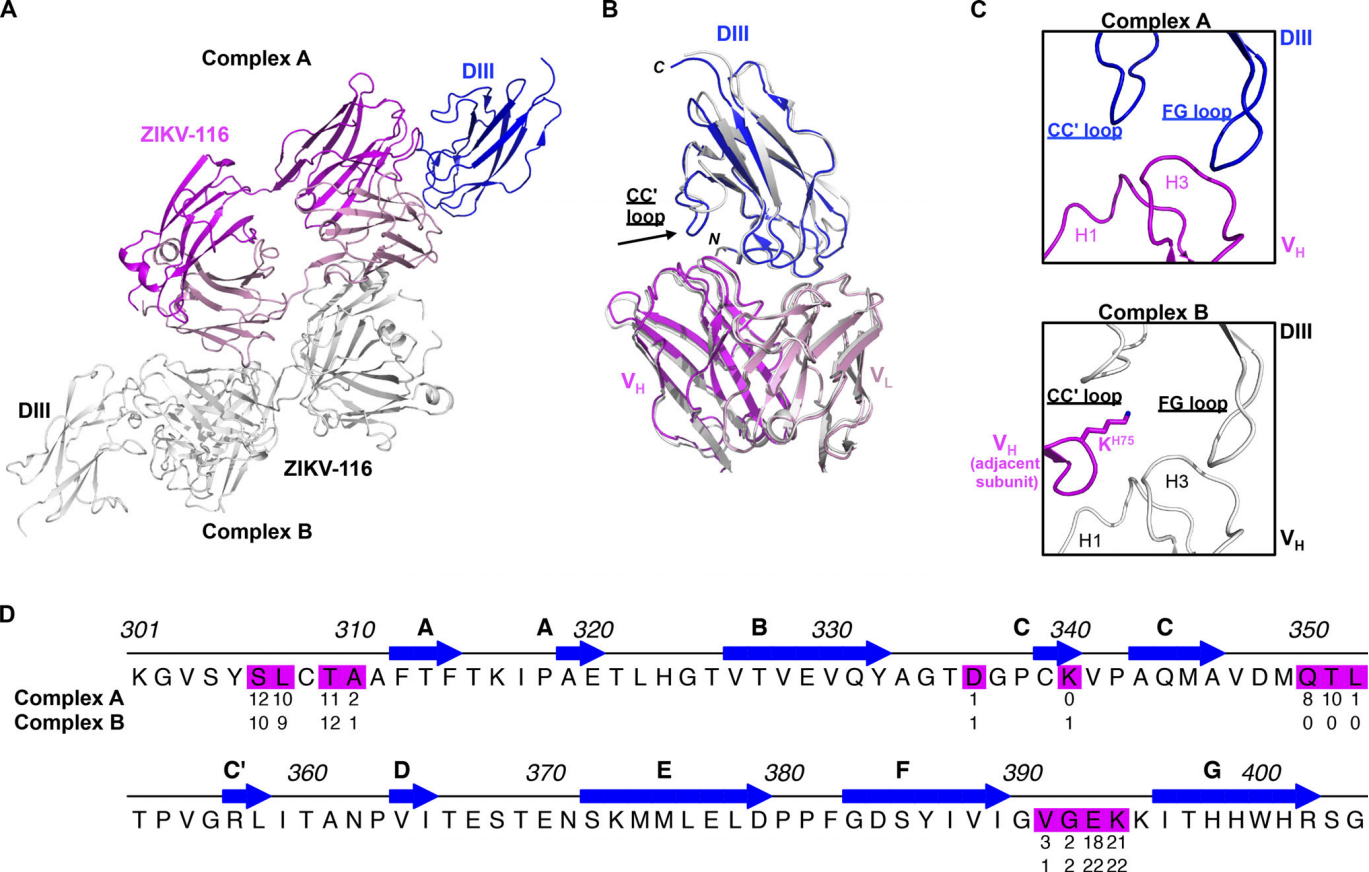
**Asymmetric unit of the ZIKV-116-DIII complex**. Related to Fig. 2. **(A)** There are two ZIKV-116-DIII complexes in one asymmetric unit of the crystal. Complex B is colored in white. In complex A, DIII is colored in blue, heavy chain in magenta, and light chain in pink. **(B)** Superimposition of DIII and variable domains of Fab between two ZIKV-116–DIII complexes. **(C)** Ribbon diagram of CC′-loop interface from complex A (upper) and complex B (down). Residue K^H75^ from adjacent asymmetric unit protruded into the ZIKV-116–DIII interface and interrupted the interaction between the CC′-loop of DIII and ZIKV-116 V_H_ in complex B. V_H_ from adjacent asymmetric unit is colored in magenta, and complex A and B are colored as above. **(D)** The DIII residues that make van der Waals contact distance <3.90 Å are colored, and the numbers below the ZIKV DIII represent the total number of contacts for each residue in each complex.

To better understand ZIKV-116–mediated neutralization, we determined the x-ray crystal structure of ZIKV-116 antigen-binding fragments (Fab) in complex with ZIKV DIII (H/PF/2013) at 2.3 Å resolution (Fig. 2 and Table S1). ZIKV-116 binds to the LR of DIII by engaging the N-terminal region as well as the BC-, CC′-, and FG-loops. The total buried surface area at the antibody-antigen interface is 1,551 Å^2^, with a shape complementarity factor of 0.71. The heavy chain accounts for 67% of the interface, with a combined buried surface of 1,042 Å^2^ (542 Å^2^ on DIII and 500 Å^2^ on the heavy chain), and the light chain contributes the remaining buried surface area (264 Å^2^ on DIII and 245 Å^2^ on the light chain). The interaction is mediated by contacts between 12 DIII residues and residues from 5 ZIKV-116 complementarity-determining regions (CDRs: (CDR-H1, CDR-H2, CDR-H3, CDR-L1, and CDR-L3) plus 2 V_H_ framework residues. Two of the DIII residues (T^E309^ and K^E394^) are contacted by both heavy and light chains. Binding of ZIKV-116 to DIII is mediated by 11 direct hydrogen bonds and 9 water-mediated networks in addition to van der Waals contacts. The N-terminal and FG-loop regions contain the majority of the interactions (79 of 99 contacts) with ZIKV-116. The FG-loop has the most extensive contact network, forming five direct hydrogen bonds and four water-mediated hydrogen bonds with ZIKV-116. The N-terminal region of DIII contributes 35 contacts and forms 5 additional direct hydrogen bonds with residues from the CDR-L3 and CDR-H2 of ZIKV-116 (Tables S2–S6). We note that there are two complexes in the crystallographic asymmetric unit that adopt overall similar conformations, with a root mean square deviation of 1.19 Å over 332 C_α_ atoms of DIII and variable domains of ZIKV-116 (Fig. S1, A and B). However, presumably due to crystal packing, we observed a different conformation of the DIII CC′-loop in one complex where it is not engaged by ZIKV-116 CDR loops but rather by an adjacent Fab (Fig. S1, C and D).

**Table S1.** Summary of crystallographic data

	ZIKV-116-DIII
**Data collection**	
Space group	P2_1_
Cell dimensions	
*a*, *b*, *c* (Å)	72.81, 89.81, 92.49
α, β, γ (°)	90.00, 103.96, 90.00
Resolution (Å)	50-2.3 (2.34–2.30)
Total reflections	325,315
Unique reflections	50,077
Redundancy	6.5 (5.1)
Completeness (%)	97.5 (94.4)
*Rmerge* (%)	9.8 (52.1)
*Rpim* (%)	4.9 (25.5)
CC1/2	1 (0.853)
*I*/σ*I*	22.5 (2.8)
Wilson B-factor (Å^2^)	42
**Refinement**	
Resolution (Å)	45–2.3 (2.34–2.30)
No. of reflections	50,016 (2,471)
*R*_work_/*R*_free_ (%)	16.7/21.1 (25.6/29.4)
No. of atoms	
Protein	8,232
Water	407
*B*-factors	
DIII	49
Fab	44
Water	45
RMS deviations	
Bond lengths (Å)	0.004
Bond angles (°)	0.66
Ramachandran plot (%)^a^	97.67/2.33/0

R_free_ was calculated with 5% of the data. Numbers in parentheses represent values in the highest-resolution shell. RMS, root mean square.

a Residues in favored, allowed, and disallowed regions of the Ramachandran plot.

**Table S2.** Van der Waals contacts for ZIKV-116–DIII complex

DIII (FIM) complex A	ZIKV-116	DIII (EHL) complex B	ZIKV-116
Ser^E306^	Ser^H56^(5), Lys^H57^(3), Tyr^H58^(4)	Ser^E306^	Ser^H56^(6), Lys^H57^(2), Tyr^H58^(2)
Leu^E307^	Ser^H56^(3), Tyr^H58^(7)	Leu^E307^	Ser^H56^(2), Tyr^H58^(7)
Thr^E309^	Asp^L92^(1), Ser^L93^(3), Tyr^L94^(2), Trp^L96^(1), Tyr^H58^(3), Val^H100A^(1)	Thr^E309^	Asp^L92^(1), Ser^L93^(4), Tyr^L94^(3), Trp^L96^(1), Tyr^H58^(3)
Ala^E310^	Asp^L92^(2)	Ala^E310^	Asp^L92^(2), Ser^L93^(1)
Asp^E336^	Tyr^L94^(1)	Asp^E336^	Tyr^L94^(1)
		Lys^E340^	Asp^H55^(1)
Gln^E350^	Tyr^H32^(1), Ser^H98^(7)		
Thr^E351^	Asn^H31^(4), Arg^H99^(6)		
Leu^E352^	Arg^H99^(1)		
Val^E391^	Arg^H99^(2), Val^H100A^(1)	Val^E391^	Arg^H99^(1)
Gly^E392^	Glu^H100C^(2)	Gly^E392^	Glu^H100C^(2)
Glu^E393^	Arg^H96^(5), Leu^H97^(3), Glu^H100C^(7), Leu^H100D^(3)	Glu^E393^	Arg^H96^(7), Leu^H97^(2), Glu^H100C^(8), Leu^H100D^(4), Tyr^L91^(1)
Lys^E394^	Trp^L32^(11), Tyr^L91^(3), Asp^L92^(3), Glu^H100C^(4)	Lys^E394^	Trp^L32^(11), Tyr^L91^(3), Asp^L92^(4), Glu^H100C^(4)

The amino acids are superscripted to indicate their specific positions in the heavy chain (H), light chain (L), or DIII (E) sequences. Interactions were determined using the ligplot program with a cutoff distance of 3.90 Å. In complex A, the heavy chain is named as chain I, light chain is named as chain M, and DIII is named as chain F. In complex B, heavy chain is named as chain H, light chain is named as chain L, and DIII is named as chain E.

**Table S3.** Van der Waals contacts summary

	CDR-H1 (26–32)	CDR-H2 (52–56)	CDR-H3 (95–102)	FRM-H	Total V_H_	CDR-L1 (24–34)	CDR-L3 (89–97)	Total V_L_
FIM complex A	5	8	42	17	72	11	16	27
EHL complex B	0	9	28	14	51	11	21	32

Interactions were determined using the ligplot program with a cutoff distance of 3.90 Å.

**Table S4.** Direct hydrogen bond contacts

DIII (FIM, complex A)	ZIKV-116	DIII (EHL, complex B)	ZIKV-116
Ser^E306^(OG)	Lys^H57^(O)		
Leu^E307^(N)	Ser^H56^(OG), Tyr^H58^(OH)	Leu^E307^(N)	Ser^H56^(OG)
Leu^E307^(O)	Tyr^H58^(OH)	Leu^E307^(O)	Tyr^H58^(OH)
Thr^E309^(OG1)	Tyr^L94^(N)	Thr^E309^(OG1)	Tyr^L94^(N)
Gln^E350^(O)	Ser^H98^(OG)		
Glu^E393^(N)	Glu^H100C^(OE1)	Glu^E393^(N)	Glu^H100C^(OE1)
Glu^E393^(OE1)	Arg^H96^(NH1)	Glu^E393^(OE1)	Arg^H96^(NH1)
Glu^E393^(OE2)	Arg^H96^(NE)	Glu^E393^(OE2)	Arg^H96^(NE)
Lys^E394^(NZ)	Glu^H100C^(OE2), Tyr^L91^(O)	Lys^E394^(NZ)	Glu^H100C^(OE2), Tyr^L91^(O)

The amino acids are superscripted to indicate their specific positions in the heavy chain (H), light chain (L) or DIII (E) sequences. Interactions were determined using ligplot program with default setting.

**Table S5.** Indirect hydrogen bond contacts

DIII (FIM, complex A)	Water	ZIKV-116	DIII (EHL, complex B)	Water	ZIKV-116
Ser^E306^(OG)	S101	Lys^H57^(O)	Ser^E306^(OG)	S179	Lys^H57^(O)
Ala^E311^(N)	S97	Asp^L92^(O)	Ala^E311^(N)	S84	Asp^L92^(O)
Thr^E335^(OG1), Thr^E335^(N)	S341	Ser^L93^(OG)	Thr^E335^(OG1), Thr^E335^(N)	S18	Ser^L93^(OG)
Val^E391^(O), Lys^E394^(N)	S6	Glu^H100C^(OE2)	Val^E391^(O), Lys^E394^(N)	S10	Glu^H100C^(OE2)
Gly^E392^(N)	S108	Arg^H99^(O)	Gly^E392^(N)	S167	Arg^H99^(O)
Glu^E393^(OE1)	S15	Tyr^L91^(OH)	Glu^E393^(OE1)	S26	Tyr^L91^(OH)
Glu^E393^(OE2)	S59	Asp^H95^(OD1)	Glu^E393^(OE2)	S144	Asp^H95^(OD1)
		Leu^H97^(N)			Leu^H97^(N)
			Ala^E333^(O)	S211	Asp^L92^(OD2)
Gln^E350^(NE2)	S156	Ser^H98^(OG, N)			
Gln^E350^(NE2)	S317	Asn^H31^(ND2)			
Gln^E350^(OE1)					

The amino acids are superscripted to indicate their specific positions in the heavy chain (H), light chain (L), or DIII (E) sequences. Interactions were determined using the ligplot program with default setting.

**Table S6.** Hydrogen bonds summary

	CDR-H1 (26-32)	CDR-H2 (52-56)	CDR-H3 (95-102)	FRM-H	Total V_H_	CDR-L3 (89-97)	Total V_L_
FIM, complex A	0+1	1+0	5+4	3+1	15	2+3	5
EHL, complex B	0+0	1+0	4+3	1+1	10	2+4	6

Hydrogen bond interactions (direct + indirect) were determined using the ligplot program with default setting.

### Variation at residue 393 modulates the binding kinetics of ZIKV-116 to DIII

We performed infection assays and found that ZIKV-116 neutralized four ZIKV Asian strains (H/PF/2013, Brazil Fortaleza, Cambodia_FSS13025, and Puerto Rico_PRVABC58) more effectively than two African strains (MR-766 and Dakar 41519; Table S7), consistent with our previous observations (Sapparapu et al., 2016). Sequence analysis of the ZIKV-116 structural epitope among the seven representative ZIKV strains showed that 11 out of 12 contact residues are invariant. However, African strains (MR-766 and Dakar 41519) have an Asp at residue 393, while most Asian strains have a Glu. Interestingly, one Asian strain (Malaysia_P6740), which has an Asp^393^ like African strains, was less effectively neutralized by ZIKV-116 compared with other Asian strains (Table S7).

**Table S7.** Neutralizing activity of ZIKV-116 and control mAb ZV-67 against different ZIKV strains

			ZIKV-116		ZV-67	
Strains	Genotype	Residue 393	IC_50_ (ng/ml)	IC_90_ (ng/ml)	IC_50_ (ng/ml)	IC_90_ (ng/ml)
H/PF/2013	Asian	E	27.4 ± 5.2	1,092 ± 396.5	236.1 ± 99.5	7,577 ± 3,558
Brazil Fortaleza	Asian	E	8.9 ± 2.6	136.7 ± 66.8	45.7 ± 4.9	1,511 ± 528.3
Cambodia FSS13025	Asian	E	19.3 ± 2.5	655.8 ± 114.3	146.4 ± 29.8	6,623 ± 715.4
Puerto Rico PRVABC58	Asian	E	8.2 ± 1.4	242.2 ± 44.0	74.6 ± 24.1	3,336 ± 877.7
Malaysia P6740	Asian	D	71.8 ± 10.8	8,435 ± 2,314	92.3 ± 15.7	4,027 ± 722.3
MR-766	African	D	365.1 ± 138.3	>100,000	104.1 ± 18.6	5,528 ± 2,824
Dakar 41519	African	D	246.9 ± 25.9	>40,000	301.9 ± 80.1	15,752 ± 5,431

The amount of mAbs that was required for 50% (IC_50_) and 90% (IC_90_) of inhibition is indicated. The indicated virus was incubated with serially diluted mAbs for 1 h at 37°C followed by addition of the mixture to Vero cells. Then, FRNT assays were performed, and the percentage of infection was calculated by comparing to no mAb–treated wells. ZIKV-116 shows greater neutralizing activity against most of ZIKV Asian strains than African strains. Control mAb ZV-67 neutralizes ZIKV Asian strains (H/PF/2013, Cambodia FSS13025, and Malaysia P6740) and African strains (MR-766 and Dakar 41519) with comparable IC_50_. The results are the average of three or more independent experiments performed in duplicate or triplicate.

**Figure 3.**
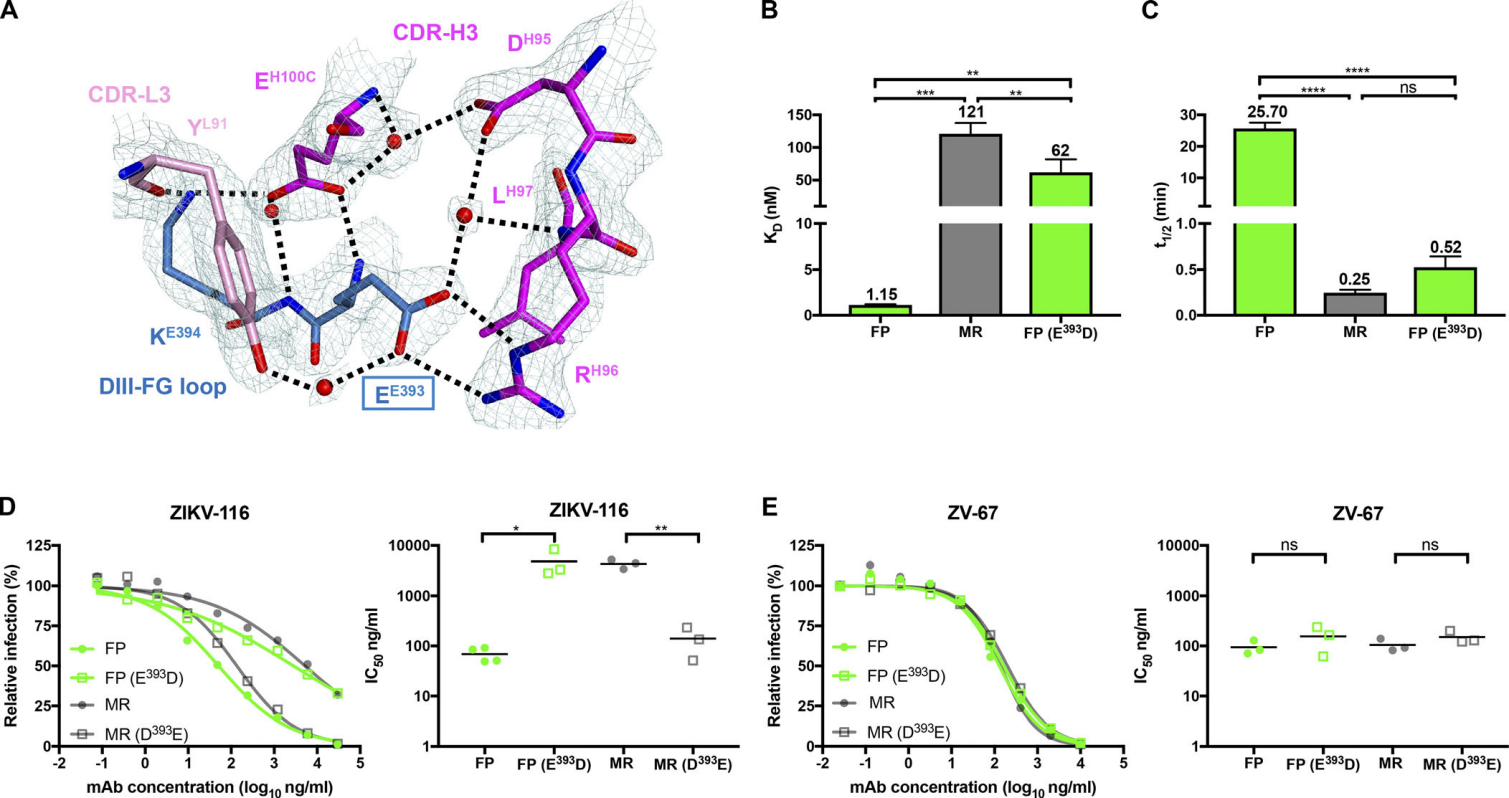
**Variation at residue 393 in the ZIKV-116 epitope is responsible for the neutralization differences between two ZIKV strains.** **(A)** Detailed interactions of DIII residues E^E393^ and K^E394^ with ZIKV-116, with *2Fo-Fc* electron density map (1.5 σ) colored in pale cyan. The water molecules are shown as spheres and colored in red, and the hydrogen bonds are marked as black dashed lines. **(B and C)** Comparison of K_D_ and *t*_1/2_ of ZIKV-116 to DIII from H/PF/2013 (FP), MR-766 (MR), and FP bearing E^393^D mutation. The results show the average of at least two independent experiments. Error bars indicate SD. **(D and E)** Neutralization profiles of mAbs against WT and mutant ZIKV FP and MR RVPs containing reciprocal amino acid substitutions at residue 393 (left). IC_50_ values from at least three independent experiments performed in duplicate (right). ZV-67 (another ZIKV DIII-LR mAb) served as a control mAb. Serial dilutions of mAbs were incubated with RVPs for 1 h at 37°C, followed by infection of Raji-DCSIGNR cells. GFP-positive, infected cells were determined by flow cytometry at 40 h. Statistical significance was determined by one-way ANOVA with a Tukey’s test (B and C) or two-tailed *t* tests (D and E). ns, not significant; *, P < 0.05; **, P < 0.01; ***, P < 0.001; ****, P < 0.0001.

In our structure, Glu^393^ is directly contacted by ZIKV-116 CDR-H3 and participates in an extensive hydrogen-bonding network, forming three direct hydrogen bonds and two water-mediated indirect hydrogen bonds with ZIKV-116 (Fig. 3 A and Tables S4–S6). To explore whether the residue 393 impacts ZIKV-116 activity, we used BLI to compare the binding of ZIKV-116 to different purified DIII proteins, including WT H/PF/2013 DIII, E^393^D H/PF/2013 DIII, and WT MR-766 DIII. ZIKV-116 bound MR-766 DIII weakly (100-fold higher K_D_ [121 nM] and *t*_1/2_ of 0.25 min) compared with H/PF/2013 DIII (Fig. 3, B and C; and Fig. 1 E). Correspondingly, the E^393^D substitution of H/PF/2013 DIII (to the residue in MR-766) significantly decreased the binding affinity and half-life, with a K_D_ of 62 nM and *t*_1/2_ of 0.52 min. Thus, it appears that a single methyl group of residue 393 in ZIKV strains can determine ZIKV-116 binding strength, most likely because the shorter side chain is incapable of optimally forming a hydrogen-bonding network with residues in CDR-H3, specifically Arg^H96^ and Glu^H100C^, which engage the DIII Glu^393^ carboxylate and main chain, respectively (Fig. 3 A).

**Figure S2.**
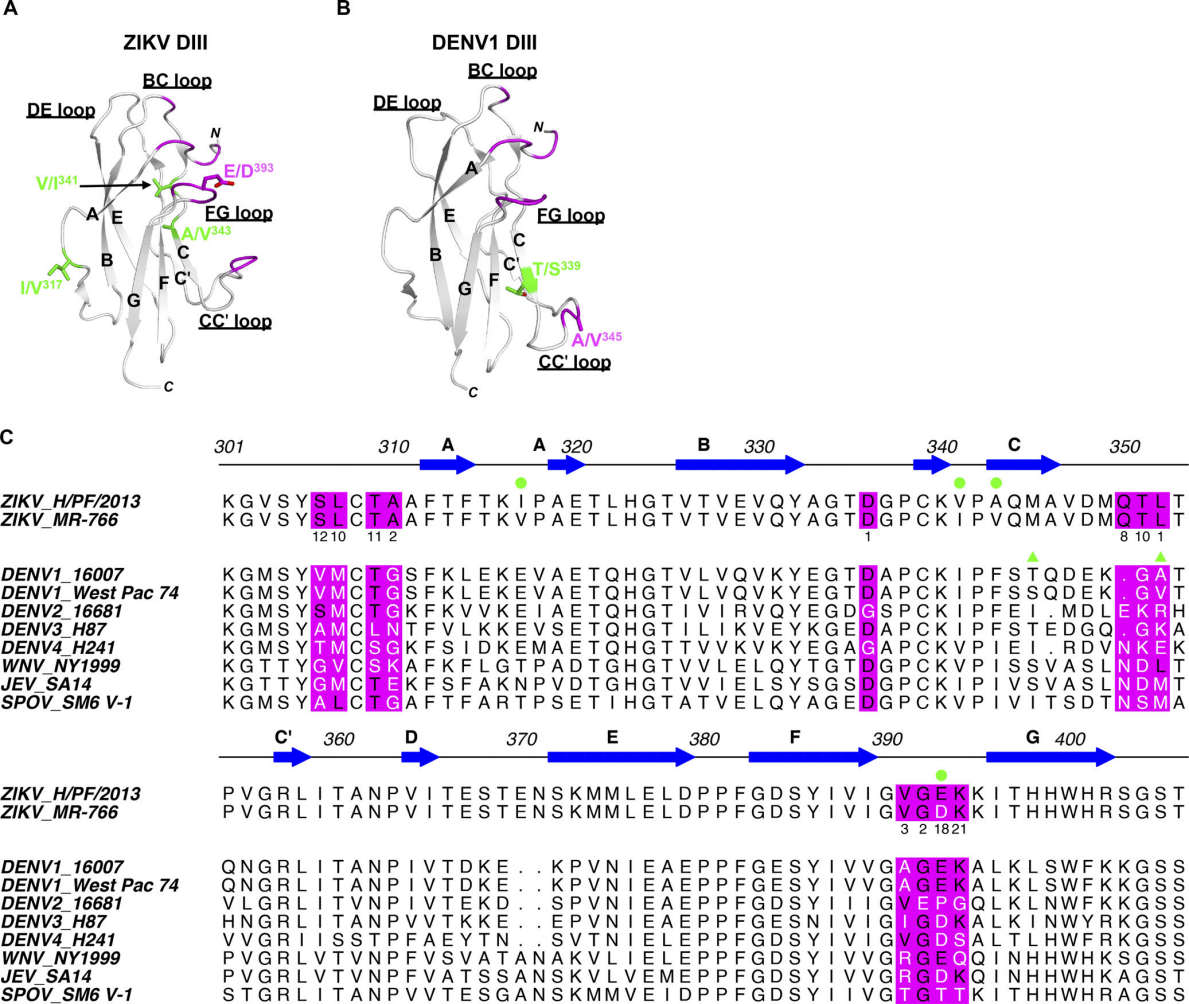
**Amino acid differences in DIII between ZIKV strains H/PF/2013 and MR-766, and DENV1 strains 16007 and West Pac-74. **Related to Fig. 5**.** **(A and B)** Ribbon diagrams of ZIKV DIII (A) and DENV1 DIII (B). ZIKV-116 epitope residues are highlighted in magenta, and variable amino acids between two ZIKV strains or two DENV1 strains are indicated by sticks. **(C)** Sequence alignment of DIII from representative flaviviruses, and ZIKV-116 epitope residues are highlighted in magenta background. Conserved residues are shown in black text, and variable residues are shown in white text within the predicted ZIKV-116 epitope. Green circles mark amino acid variations between ZIKV H/PF/2013 and MR-766. Green triangles mark amino acid variations between DENV1 strains 16007 and West Pac-74.

The amount of ZIKV-116 required to neutralize Malaysia_P6740 strain is higher than that required to inhibit Asian strain H/PF/2013, and lower than the amount required to neutralize African strains (Table S7). Consistently, the DIII^FP(E393D)^ mutant, which is identical to the naturally occurring DIII of Malaysia_P6740 strain, bound ZIKV-116 with an ∼50-fold lower affinity than DIII^FP^ and ∼2-fold higher affinity than WT DIII^MR^. While the structurally defined epitope is strictly conserved among analyzed ZIKV strains with the exception of residue 393, the African MR-766 strain varies at three additional positions in DIII regions that could distally affect the epitope and thereby mAb binding (Fig. S2).

### Variation at residue 393 modulates the neutralizing activity of ZIKV-116 against different ZIKV strains

To assess the role of residue 393 in differential ZIKV-116 neutralization, we generated ZIKV reporter virus particles (RVPs) with H/PF/2013 and MR-766 structural proteins and incorporated the reciprocal changes at residue 393. Exchanging the amino acid at residue 393 dramatically reversed the ZIKV-116 neutralization phenotype between the H/PF/2013 and MR-766 strains. The IC_50_ values of WT H/PF/2013 and E^393^D variant increased from 69 to 4,855 ng/ml, making the neutralization of E^393^D H/PF/2013 RVPs similar to the neutralization of WT MR-766. Reciprocally, D^393^E MR-766 RVPs were neutralized by ZIKV-116 similarly to WT H/PF/2013 RVPs (approximately twofold difference in IC_50_; Fig. 3 D). In comparison, the presence of D^393^ or E^393^ had little effect on mAb ZV-67, which binds an overlapping DIII-LR epitope and neutralizes H/PF/2013 and MR-766 strains equivalently (Fig. 3 E; Zhao et al., 2016). Taken together with our binding studies, these observations strongly suggest that differential ZIKV neutralization by ZIKV-116 is primarily controlled by residue 393 variation, and that mAb-binding affinity correlates well with neutralization potency against distinct ZIKV strains.

### Differential neutralization of DENV1 genotypes

**Figure 4.**
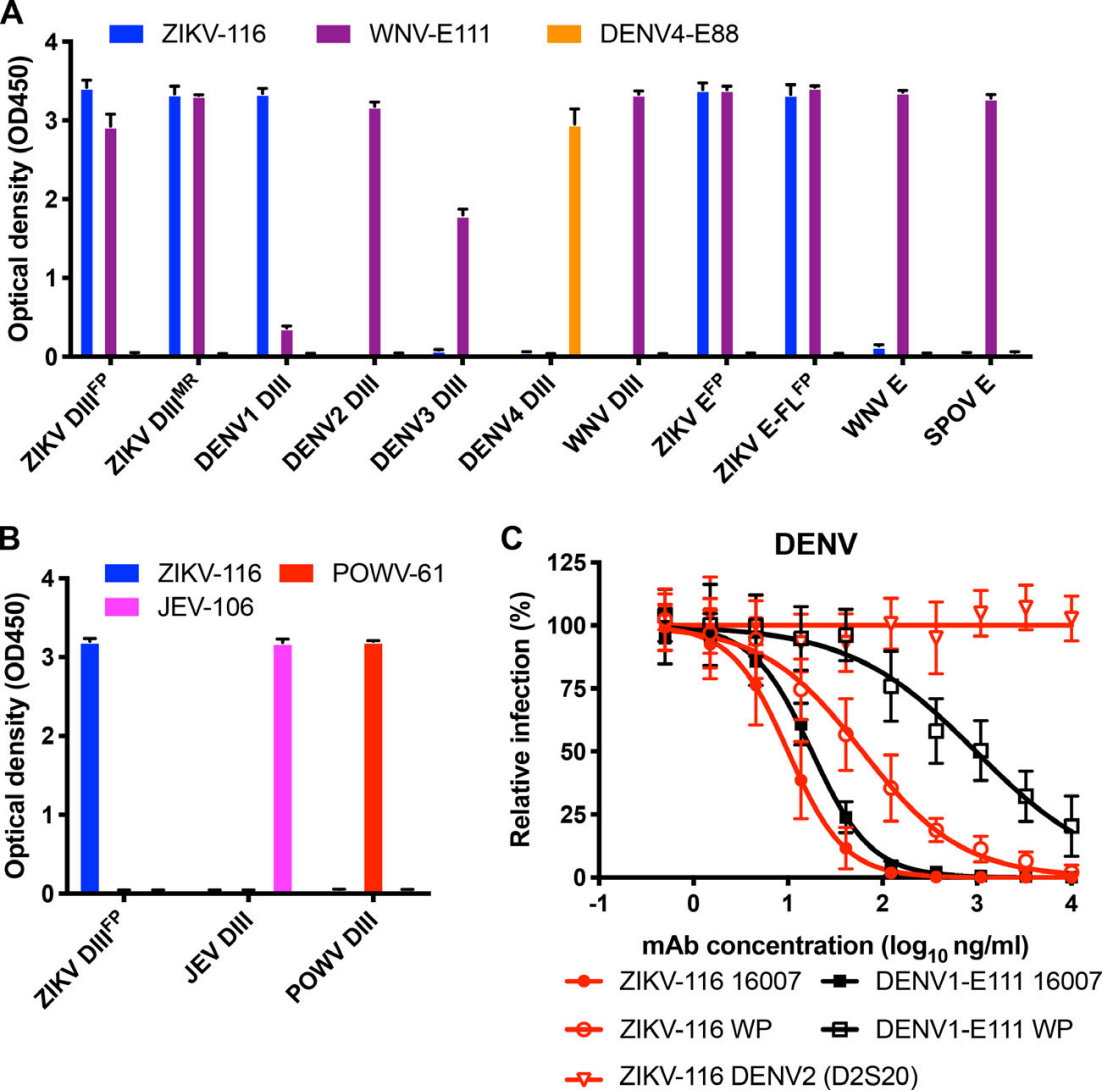
**ZIKV-116 neutralizes DENV1 strain 16007 better than West Pac-74**. **(A)** ZIKV-116 or control mAbs (WNV-E111 [flavivirus cross-reactive] and DENV4-E88 [DENV4 type-specific]) were tested for binding to the indicated flavivirus proteins (ZIKV E, ZIKV E-FL [mutant strain of H/PF/2013], ZIKV DIII [strains of H/PF/2013 and MR-766], WNV E and DIII, SPOV E [Spondweni virus], and DENV1–4 DIII) as indicated in ELISA. **(B)** ZIKV-116 or control mAbs (JEV-106 [JEV DIII-specific] and POWV-61 [Powassan DIII-specific]) were tested for binding to the indicated DIII of ZIKV H/PF/2013, JEV, and POWV. The results are representative of two independent experiments performed in triplicate. **(C)** Neutralization curves of DENV2 (D2S20) and DENV1 (16007 and West Pac-74 [WP]) by ZIKV-116 and control mAb DENV1-E111. The indicated virus was incubated with serial-diluted mAbs for 1 h at 37°C followed by addition of the mixture to Vero cells. Then, FRNT assays were performed, and the percentage of infection was calculated by comparing to no mAb–treated wells. Data were pooled from two or more independent experiments performed in triplicate or quadruplicate.

**Figure 5.**
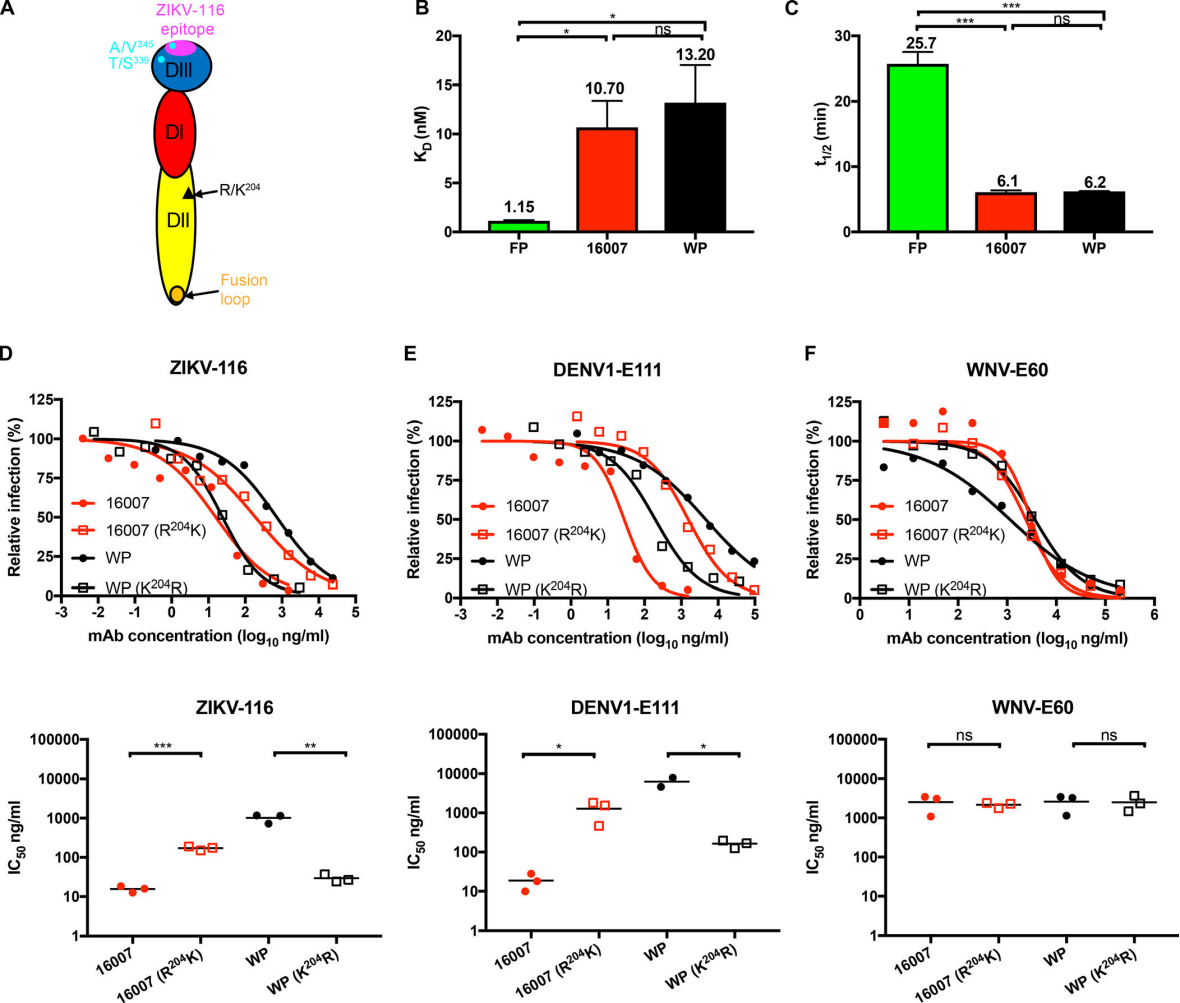
**Variation at residue 204 in DII is responsible for the neutralization difference between two DENV1 strains.** **(A)** Cartoon of DENV1 E protein (DI, red; DII, yellow; DIII, blue). ZIKV-116 epitope is highlighted in magenta. Amino acid differences (339 and 345) between 16007 and West Pac-74 (WP) DIII are shown in cyan. Residue 204, which is responsible for the difference in neutralization between two DENV1 strains, is marked as a black triangle in DII. **(B and C)** Comparison of K_D_ and *t*_1/2_ of ZIKV-116 to DIII from H/PF/2013, 16007, and West Pac-74. The results from at least two independent experiments are shown. Error bars represent SD. **(D–F)** Effects of residue 204 on ZIKV-116 neutralization of DENV1 strains 16007 and West Pac-74. Neutralization profiles of mAbs against WT DENV1 strains 16007 and West Pac-74 RVPs compared with mutant RVPs containing reciprocal amino acid at residue 204 (upper). IC_50_ values are from three independent experiments (bottom). DENV1-E111, which was reported to display differential neutralization against DENV1 genotypes, is used as a positive control mAb, and WNV-E60 serves as a negative control mAb. Statistical significance was determined by one-way ANOVA with a Tukey’s test (B and C) or two-tailed *t* tests (D–F). ns, not significant; *, P < 0.05; **, P < 0.01; ***, P < 0.001. FP, H/PF/2013.

Sequence analysis of the ZIKV-116 mAb showed that it used the V_H_3–23 heavy chain and V_K_1–5 light chain genes, and V_H_3–23/V_K_1–5 mAbs against ZIKV have been reported to cross-react with DENV1 (Sapparapu et al., 2016; Robbiani et al., 2017; Magnani et al., 2017; Niu et al., 2019). To investigate cross-reactivity, we tested the specificity of ZIKV-116 against a panel of recombinant proteins from different flaviviruses by ELISA (Fig. 4, A and B). In this qualitative assay, ZIKV-116 recognized H/PF/2013 DIII, E, and E-FL (a fusion loop mutant) equally well as MR-766 DIII. We also found that ZIKV-116 can recognize DIII from DENV1, but not DIII from other DENV serotypes (DENV2, DENV3, or DENV4) or E or DIII proteins from other flaviviruses (WNV, JEV, Spondweni, or Powassan viruses). Based on these binding properties, we assessed whether ZIKV-116 could inhibit infection of different DENV1 strains. We found that ZIKV-116 neutralized a DENV1 genotype 2 strain (16007) more effectively than a genotype 4 strain (West Pac-74), with IC_50_ values of 11 and 73 ng/ml, respectively (Fig. 4 C). No neutralization activity was observed to DENV2. Sequence comparison revealed only two amino acid differences in DIII between 16007 and West Pac-74, both located in the CC′-loop (T^339^S and A^345^V). Residue 345 but not 339 is located within the predicted ZIKV-116 footprint on DENV1 DIII (Fig. 5 A and Fig. S2).

### A residue distal to the ZIKV-116 epitope modulates DENV1 strain neutralization sensitivity

Since ZIKV-116 less efficiently neutralized DENV1 West Pac-74 than 16007, we evaluated the binding affinity of ZIKV-116 to DIII from these two viruses. BLI experiments revealed that ZIKV-116 bound DIII from the two strains with nearly identical affinities and kinetics, with a K_D_ of 10.7 nM and *t*_1/2_ of 6.1 min for DIII^16007^ versus a K_D_ of 13.2 nM and *t*_1/2_ of 6.2 min for DIII^West Pac-74^ (Fig. 5, B and C); these data are consistent with the complete conservation of the predicted ZIKV-116 epitope on DENV1 besides the identified A^345^V variation (Fig. S2). We previously reported a DENV1-specific mAb that recognizes a cryptic epitope in DIII and exhibited genotypic differences in neutralization, DENV1-E111 (Austin et al., 2012; Fig. 4 C). Dowd et al. (2015) went on to discover that a DII residue distal from the epitope (residue 204; Fig. 5, A and E) altered DENV1-E111 neutralization, perhaps by changing the display of the DIII epitope in a genotype-dependent manner. To investigate whether this same residue 204 variation also accounts for the different neutralization activity of ZIKV-116, we performed neutralization assays using DENV1 16007 and West Pac-74 RVPs bearing reciprocal changes at E residue 204. Exchange of K^204^R on the West Pac-74 background shifted the neutralization curves to that seen with WT 16007 RVPs (IC_50_ of 29 ng/ml for K^204^R West Pac-74 versus 16 ng/ml for WT 16007). Reciprocally, exchange of R^204^K on the 16007 background led to an 11-fold reduced ZIKV-116 neutralization potency (Fig. 5 D). In contrast, a control mAb, WNV-E60, which binds the E-FL epitope in DII, showed similar neutralization potency with all WT and variant DENV1 RVPs (Fig. 5 F).

### A reverted, unmutated ancestor form of ZIKV-116 is capable of binding and neutralizing ZIKV and DENV1 strains

**Figure 6.**
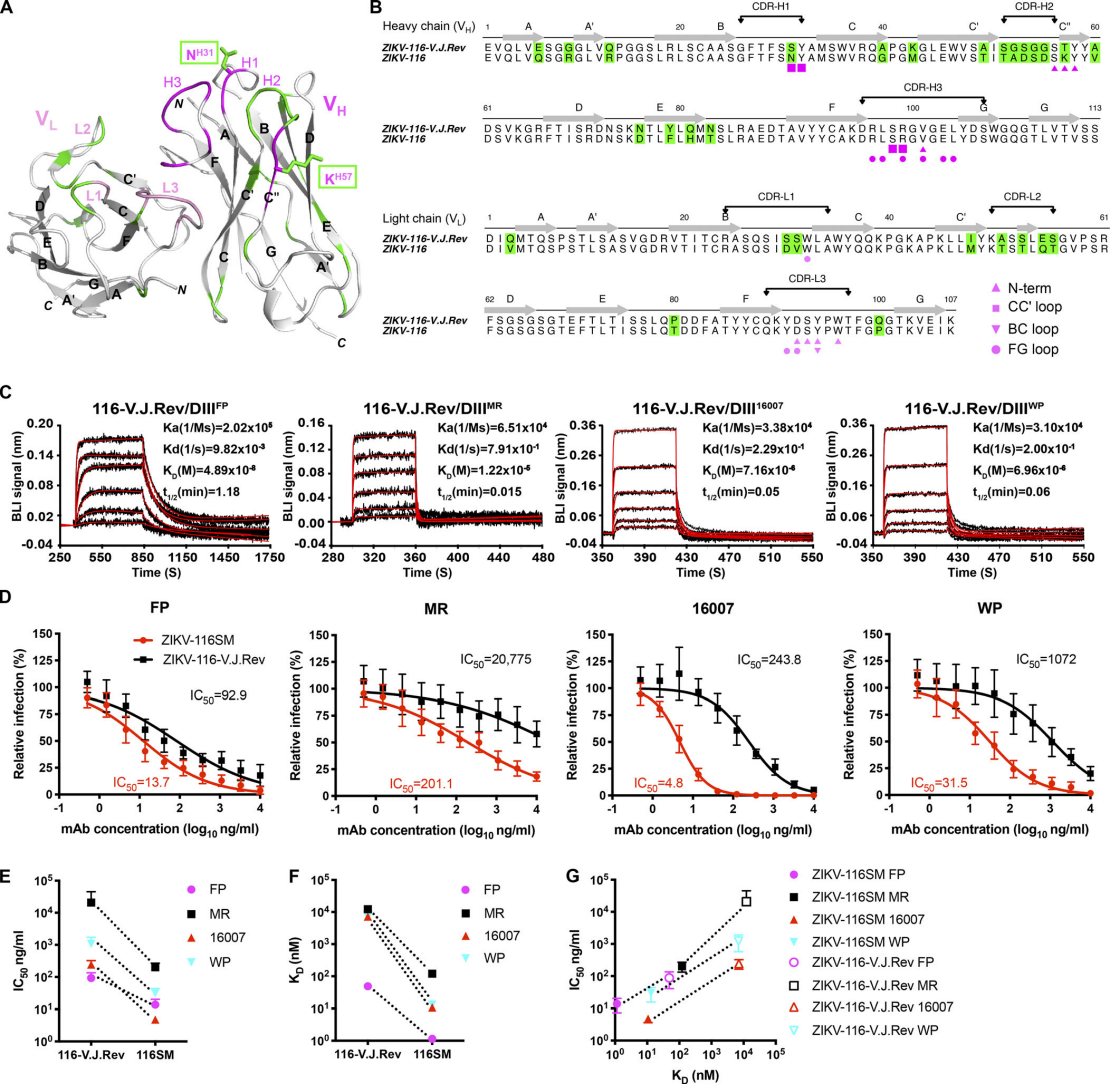
**ZIKV-116-V.J.Rev has reduced affinity and neutralization potency to ZIKV and DENV1**. **(A)** Ribbon diagrams of variable region of ZIKV-116 mAb, with the backbone of contacts highlighted in pink for light chain variable domain (V_L_) and magenta for V_H_. Somatic mutation residues are colored in green. The substituted residues that are involved with DIII contacts are drawn as sticks. **(B)** Amino acid sequence alignment of the V_H_ and V_L_ of ZIKV-116 to its V-J reverted germline genes. Somatic mutations are shaded as in A. Residues marked by magenta/pink symbols below the sequence show amino acids engaged in the interface with DIII. **(C)** Analysis of the binding of ZIKV-116-V.J.Rev to DIII from ZIKV H/PF/2013, ZIKV MR-766, DENV1 16007, and DENV1 West Pac-74 as measured by BLI. The experimental curves (black lines) were fitted using a 1:1 Langmuir binding analysis (red lines), and the results are representative of two or three independent experiments. **(D)** Neutralization profiles of ZIKV-116 and ZIKV-116-V.J.Rev against indicated ZIKV and DENV1 strains as assessed by FRNT on Vero cells. The data are pooled results from at least three independent experiments performed in triplicate or quadruplicate. **(E and F)** IC_50_ values and K_D_ (nM) comparison between somatically mutated (ZIKV-116SM) and V-J reverted germline version (ZIKV-116-V.J.Rev). **(G)** Neutralization potency (IC_50_ values) of four strains by ZIKV-116SM and ZIKV-116-V.J.Rev versus K_D_ to DIII of ZIKV H/PF/2013, ZIKV MR-766, DENV1 16007, and DENV1 West Pac-74. FP, H/PF/2013; MR, MR-766.

The affinity-matured ZIKV-116 is encoded by V_H_3–23 and V_K_1–5 germline genes, with 18 aa substitutions in the V_H_ domain and 10 aa substitutions in the V_L_ domain. Only 2 of the 28 residues mutated from V and J gene segments are part of the ZIKV-116 paratope, S^H31^N in CDR-H1 and T^H57^K adjacent to CDR-H2, and both residues make only modest contacts with the DIII-LR epitope (Tables S2–S6). The remaining 26 residue substitutions are located mainly in the V_H_ framework regions, CDR-H2 and CDR-L1/2 (Fig. 6, A and B). To investigate the properties of the ZIKV-116 clonal lineage, we reverted ZIKV-116 to the inferred germline sequence (reverted, unmutated ancestor mAb, ZIKV-116-V.J.Rev) and produced it using the same IgG1 expression vectors as the affinity-matured mAb. Both the V and J gene segments were reverted to the respective templated germline sequences. The reverted antibody retained the original CDR-H3 of the mature WT ZIKV-116 antibody, which possesses one somatically mutated residue when compared with the inferred germline D gene segment IGHD3-10*01.

We next evaluated the binding of ZIKV and DENV1 DIII proteins to immobilized ZIKV-116-V.J.Rev using BLI (Fig. 6 C). Surprisingly, ZIKV-116-V.J.Rev showed only a moderately decreased binding to ZIKV H/PF/2013 DIII (K_D_ of 48.9 nM and *t*_1/2_ of 1.18 min). On the other hand, binding to DIII from ZIKV MR-766 and the two DENV1 strains was markedly weaker (K_D_ of ∼10 µM for DIII^MR-766^ and K_D_ of ∼7 µM for DIII^16007^ and DIII^West Pac-74^). The decreased binding to DIII of all four viruses was due primarily to increased dissociation rates (Fig. 6 C and Table S8).

**Table S8.** BLI results for ZIKV and DENV1 DIII binding to mAbs

mAbs	DIII	Binding parameters^a^					
		k_a_ (10^5^ M^−1^ s^−1^)	k_d_ (10^−3^ s^−1^)	K_D_ (nM), kinetic	K_D_ (nM), equilibrium^b^		*t*_1/2_ (min)^c^
ZIKV-116	DIII^H/PF/2013^ (pH 5.5)	3.27 ± 0.29	0.73 ± 0.13	2.24 ± 0.19	5.05 ± 0.41	16.06 ± 2.62	
ZIKV-116	DIII^H/PF/2013^	3.94 ± 0.14	0.45 ± 0.03	1.15 ± 0.07	7.54 ±1.56	25.7 ± 1.84	
ZIKV-116	DIII^MR-766^	3.91 ± 0.26	47.17 ± 6.40	121 ± 16.52	141 ± 22.34	0.25 ± 0.03	
ZIKV-116	DIII^H/PF/2013^ (E^393^D)	3.76 ± 0.52	22.83 ± 5.28	62 ± 19.93	68.67 ± 9.32	0.52 ± 0.12	
ZIKV-116	DIII^16007^	1.84 ± 0.54	1.90 ± 0.08	10.7 ± 2.70	15.95 ± 6.72	6.10 ± 0.25	
ZIKV-116	DIII^West Pac-74^	1.47 ± 0.41	1.86 ± 0.01	13.2 ± 3.82	16.75 ± 3.89	6.21 ± 0.04	
ZIKV-116 V.J.Rev	DIII^H/PF/2013^	2.02 ± 0.20	9.82 ± 0.10	48.9 ± 5.37	68.15 ± 17.18	1.18 ± 0.01	
ZIKV-116 V.J.Rev	DIII^MR-766^	0.65 ± 0.18	790.7 ± 194.6	12,233 ± 986.6	16,267 ± 2,479	0.015 ± 0.004	
ZIKV-116 V.J.Rev	DIII^16007^	0.34 ± 0.12	229.0 ± 9.90	7,160 ± 2,220	39,050 ± 34,578	0.05 ± 0.002	
ZIKV-116 V.J.Rev	DIII^West Pac-74^	0.31 ± 0.09	199.5 ± 48.79	6,955 ± 3557	48,300 ± 41,861	0.065 ± 0.01	

a Values are means ± SD from at least two independent experiments.

^b^ K_D_, equilibrium was determined as described in the Materials and methods.

c Calculated from the dissociation constant: *t*_1/2_ = ln(2)/k_d_.

Neutralization assays revealed that ZIKV-116-V.J.Rev has potent activity against ZIKV H/PF/2013, only a sevenfold decrease compared with ZIKV-116 (Fig. 6 D). The V-J–reverted mAb was also capable of neutralizing the two DENV1 strains, albeit with 30- to 50-fold lower activity compared with the somatically mutated mAb. However, ZIKV-116-V.J.Rev neutralization of ZIKV MR-766 was only modest, with an apparent 100-fold reduced activity that failed to block infection of 50% of the African virus even at the highest mAb concentration tested. We note that the binding and neutralization activity to all ZIKV and DENV strains increased during somatic evolution of the ZIKV-116 lineage (Fig. 6, E and F). Further, the increased neutralization potency of the affinity-matured mAb relative to the germline configuration is highly correlated for each virus with the monotypic binding affinity we measured for DIII (Fig. 6 G).

### The ZIKV-116 epitope is not fully exposed in the cryo-EM model of mature ZIKV

**Figure 7.**
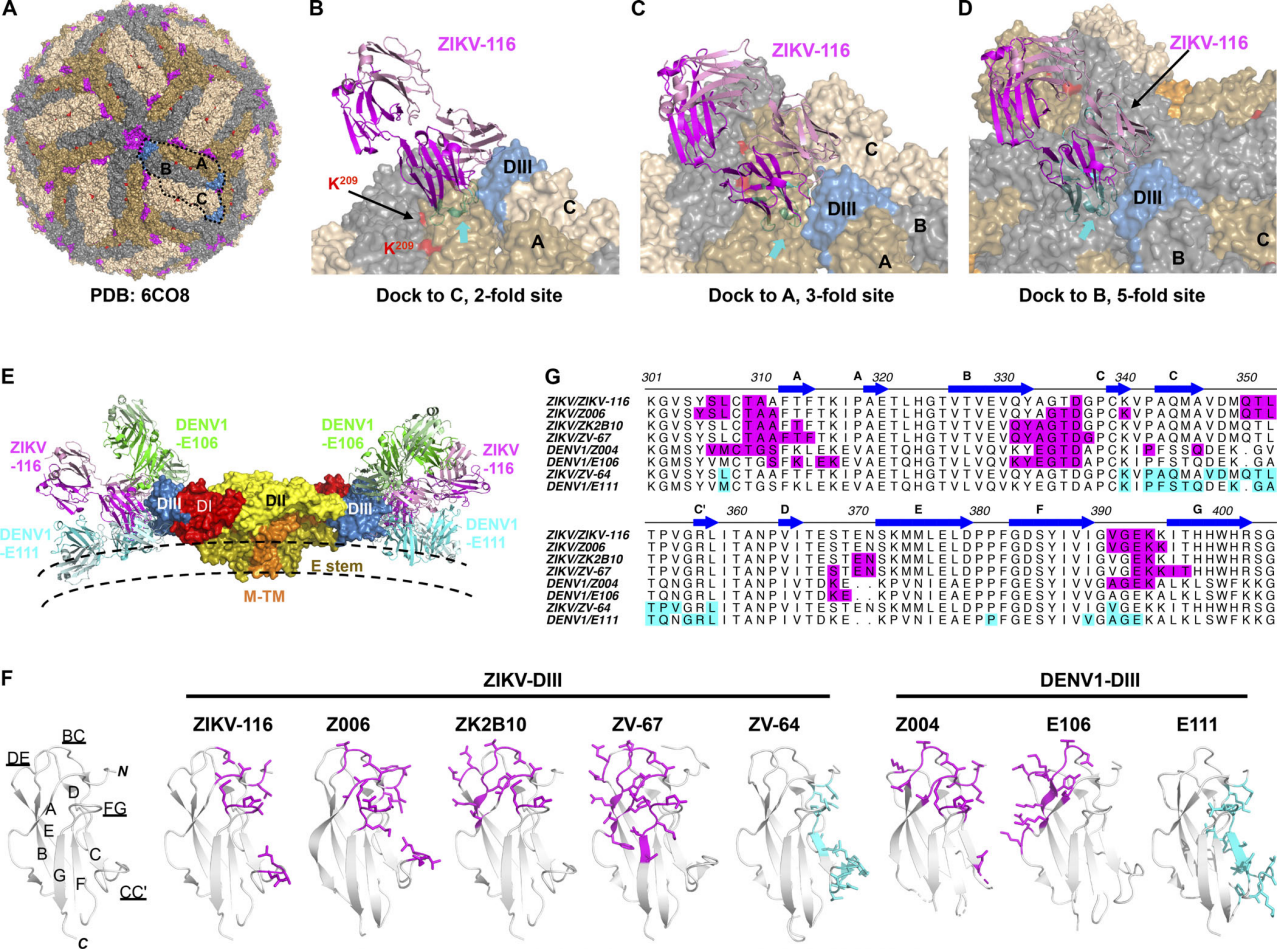
**Epitope comparison of neutralizing DIII-specific mAbs against ZIKV and DENV1**. **(A)** Mapping of the ZIKV-116 epitope onto the mature ZIKV virion (PDB 6CO8). E proteins in three symmetries are colored in wheat (twofold; C), olive (threefold; A), and gray (fivefold; B). The ZIKV-116 epitope is colored in magenta, and K^209(ZIKV)^ correlating to R/K^204(DENV1)^ is colored in red. **(B–D)** Docking of the ZIKV-116 Fab onto representative twofold (B), threefold (C), and fivefold (D) sites. Clashes with adjacent E proteins were highlighted in cyan and indicated with arrows. **(E)** Docking of the ZIKV-116, DENV1-E106 (exposed A-strand epitope), and DENV1-E111 (cryptic CC′-loop epitope) Fabs onto the M-E dimer of the mature virion. ZIKV-116 binding to the LR of DIII is located between the positions of DENV1-E106 and DENV1-E111. **(F)** Ribbon diagrams of DIII, and epitopes recognized by mAbs are rendered as sticks. LR/A-strand epitopes are colored in magenta, and CC′-loop epitope is colored in cyan. **(G)** Sequence alignment of ZIKV and DENV1 with highlighted antibody epitopes (same coloring as in F). ZIKV-Z006 (PDB 5VIG), ZIKV-ZK2B10 (PDB 6JEP), ZIKV-ZV-67 (PDB 5KVG), DENV1-Z004 (PDB 5VIC), DENV1-E106 (PDB 4L5F), ZIKV-ZV-64 (PDB 5KVF), and DENV1-E111 (PDB 4FFY) were used for the analysis.

**Figure S3.**
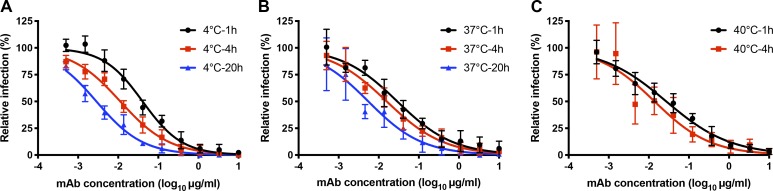
**Time and temperature dependence of ZIKV inhibition.** **(A–C)** ZIKV H/PF/2013 was incubated with ZIKV-116 or media for 1, 4, and 20 h at 4°C and 37°C (A and B) or 1 and 4 h at 40°C (C). Virus-mAbs or viruses alone were added to Vero cells after incubation, and infectivity was assessed 40 h later using an FFA assay. The relative infection was calculated by comparing no mAb-treated wells at each incubation time for each temperature. Data shown are pooled from two independent experiments in duplicate or triplicate. Error bars indicate SD.

Three cryo-EM structures of mature ZIKV H/PF/2013 have been reported to date (Sirohi et al., 2016; Kostyuchenko et al., 2016; Sevvana et al., 2018), and we used the highest-resolution model to evaluate how ZIKV-116 might interact with the virion (Protein Data Bank [PDB] 6CO8). Analysis of the virion structure indicates that the DIII-LR epitope is predominantly solvent exposed, with the exception of some parts of the CC′-loop (Fig. 7 A). However, docking of our ZIKV-116-DIII complex to all three unique T = 3 environments on the virus revealed that steric hindrance imposed by adjacent E subunits would cause clashes with the ZIKV-116 V_H_ domain (Fig. 7, B–D). Thus, the ZIKV-116 epitope can be considered to be partially cryptic in the context of the mature, icosahedrally averaged structure. In fact, the predicted virion binding orientation of ZIKV-116 places the Fab lying nearly parallel to the virus membrane, similar to a typical CC′-loop engaging mAb (i.e., DENV1-E111; Austin et al., 2012) and in contrast to some other DIII-LR mAbs, which project away from the virion surface (i.e., DENV1-E106; Edeling et al., 2014; Fig. 7, E–G). We speculate that as claimed for other viruses (e.g., WNV, DENV, norovirus, HIV, and hepatitis C virus; Dowd et al., 2011; Sukupolvi-Petty et al., 2013; Lindesmith et al., 2014; Keck et al., 2016; Mengistu et al., 2015), dynamic motions or “breathing” of virions might enable cryptic epitopes to become transiently exposed, allowing for antibodies like ZIKV-116 to bind and neutralize infection. Virion breathing and its effects on epitope accessibility have been well studied with other flaviviruses, and time-dependent increases in mAb neutralization have been observed for mAbs that recognize cryptic epitopes (Austin et al., 2012; Dowd et al., 2011; Li et al., 2018; Lok et al., 2008). To experimentally address this issue, we evaluated the time and temperature dependence of ZIKV-116 neutralization (Fig. S3). We observed mildly enhanced ZIKV neutralization by ZIKV-116 following prolonged incubation times, although these changes do not appear particularly temperature dependent.

## Discussion

Previous studies have shown that antibody-mediated immune responses against flaviviruses are protective (Nybakken et al., 2005; Sapparapu et al., 2016; Shrestha et al., 2010; Fernandez et al., 2018). The identification of the mechanisms of neutralization and key epitopes of potently neutralizing antibodies has implications for antibody-based therapies and vaccine development. Here, using structural and functional approaches, we characterized a DIII-LR mAb that is commonly elicited in humans against ZIKV and found to cross-react with DENV1, and determined the molecular basis of its inhibitory activities.

Mechanism-of-action experiments showed that ZIKV-116 could efficiently block infection even after the virus has attached to target cells. Indeed, ZIKV-116 could block viral fusion with cell membranes using an in vitro assay. We and others have previously described DIII-LR mAbs against WNV (Thompson et al., 2009), JEV (Fernandez et al., 2018), and ZIKV (Wang et al., 2019) that are capable of blocking viral membrane fusion, and similar mechanisms of action have been reported for flavivirus mAbs against other epitopes (Gollins and Porterfield, 1986; Füzik et al., 2018; Zhang et al., 2016; Vogt et al., 2009; Zhang et al., 2015; Cockburn et al., 2012). Consistent with its ability to block fusion, ZIKV-116 retained high affinity binding to DIII at an acidified endosomal pH. Fusion inhibition may be a particularly important neutralization mechanism for flavivirus mAbs since virions can apparently enter target cells through multiple receptors and entry pathways (Pokidysheva et al., 2006; Hamel et al., 2015; Sirohi and Kuhn, 2017; Perera-Lecoin et al., 2013; Cruz-Oliveira et al., 2015).

We found that the neutralization potency of ZIKV-116 to different ZIKV strains is highly correlated with the identity of residue 393 in the FG-loop of DIII. Using engineered reporter viruses, we found that an E^393^D change in the Asian ZIKV H/PF/2013 strain resulted in significantly diminished neutralization, while the reciprocal D^393^E substitution in the African ZIKV MR-766 strain dramatically improved neutralization. These results support the idea that E^393^ is critical for optimal ZIKV-116 engagement, and indeed, neutralization is highly correlated with ZIKV DIII binding affinity and half-life. We previously described E^393^ as a contact residue for a murine mAb, ZV-67 (Zhao et al., 2016), which also engages the DIII-LR epitope, albeit from a different orientation compared with ZIKV-116. However, ZV-67 neutralized all examined ZIKV strains equivalently, and activity is unperturbed by E^393^D substitutions. The likely explanation for this disparity is that E^393^ coordinates an elaborate hydrogen-bonding network at the ZIKV-116 paratope–epitope interface while it is a minor player in the murine mAb interface.

**Figure S4.**
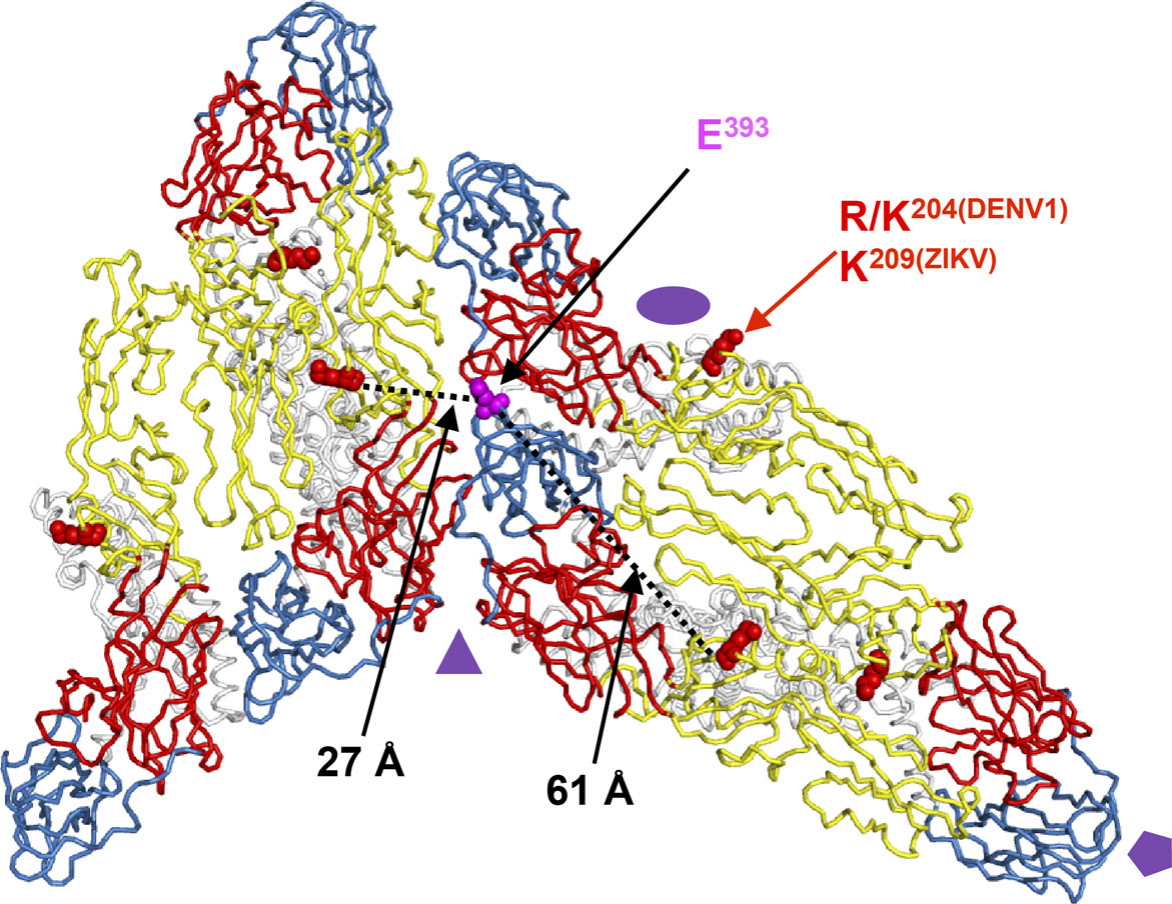
**Residue 204^DENV1^/209^ZIKV^ is located distally from the structural ZIKV-116 epitope.** Two representative asymmetric units of the mature ZIKV virion (PDB 6CO8) are shown as ribbon structures (DI, red; DII, yellow; DIII, blue; transmembrane domain, white). The icosahedral symmetry axes are indicated by a purple oval (twofold), triangle (threefold), and pentagon (fivefold). Red spheres mark residue R/K^204(DENV1)^/K^209(ZIKV)^, and magenta spheres mark residue E^393^. The distance between the C_α_ of two residues are measured using PyMOL software. E^393^ is ∼60 Å away from residue R/K^204(DENV1)^ on the E monomer, and the closest distance of E^393^ to R/K^204(DENV1)^ from adjacent E monomer is ∼30 Å.

Our studies have revealed that ZIKV-116 can neutralize DENV1, with higher potency measured for genotype 2 strain 16007 relative to genotype 4 strain West Pac-74. Quantitative binding experiments showed that DIII from both viruses binds ZIKV-116 equivalently. Similar differences in genotype-dependent neutralization of DENV1 were observed for the murine mAb DENV1-E111, which primarily recognizes a cryptic DIII CC′-loop epitope (Austin et al., 2012; Dowd et al., 2015). For DENV1-E111 the genotype-dependent differences in neutralization were found to be due in part to DII residue 204, which may allosterically affect the display of DIII epitopes (Fig. 5 E; Dowd et al., 2015). Here, we have found that residue 204 plays a similar role in modulating ZIKV-116 neutralization sensitivity of DENV1 strains, with R^204^-containing viruses better inhibited than K^204^ viruses. Similar to DENV1-E111, our analysis suggests that the DIII-LR epitope engaged by ZIKV-116 is cryptic. Indeed, while the epitope is predominantly solvent exposed, docking of our crystal structure onto cryo-EM models of mature viruses indicated steric clashes of the Fab that could only be resolved by envelope rearrangements. While it is tempting to speculate that DENV1 residue 204 could play a role in such virion conformational dynamics, as it is located ∼60 Å away from the DIII-LR epitope of the same E protein, it is formally possible that it is instead a direct contact residue, as it is located only ∼30 Å distant by icosahedral symmetry (Fig. S4). The precise role of residue 204 may be best addressed in future studies of virion-antibody complexes using cryo-EM.

Human mAbs that target DIII and cross-react between ZIKV and DENV1 have been described from at least eight donors examined by four independent groups (Robbiani et al., 2017; Sapparapu et al., 2016; Magnani et al., 2017; Niu et al., 2019). Remarkably, all of these mAbs use the same germline precursors as ZIKV-116, specifically the V_H_3–23 heavy chain and V_K_1–5 light chain, arguing strongly that this pairing represents a broadly shared, public antibody lineage. Similar to ZIKV-116, many of these V_H_3–23/V_K_1–5–derived mAbs exhibit better neutralization of Asian ZIKV strains relative to African strains (Niu et al., 2019; Magnani et al., 2017). Further, protection studies in macaques with one of these mAbs, Z004, led to a viral escape mutant with an E^393^D substitution (Keeffe et al., 2018), a result supporting our conclusion that natural variation at residue 393 determines the differential neutralization of ZIKV strains by ZIKV-116. In recent years, germline-targeted vaccine development has been explored for HIV, with immunogens engineered for optimal engagement of broadly neutralizing mAb precursors (Jardine et al., 2016; Steichen et al., 2016; Duan et al., 2018; Burton, 2019). We found that the V-J reverted germline form of ZIKV-116 can broadly bind and neutralize ZIKV and DENV1 strains, with the highest potency against Asian ZIKV strain H/PF/2013. Thus, to induce V_H_3–23/V_K_1–5 lineage mAbs like ZIKV-116, vaccine strategies should probably include ZIKV E proteins that encode E^393^, although suboptimal protection against African ZIKV strains is possible.

In summary, we have defined the structural and molecular mechanism of a representative DIII-LR–specific mAb from a common germline lineage in humans. We found that the potency of ZIKV-116 against ZIKV strains is tightly correlated with a single epitope residue that modulates binding affinity, while differential DENV1 strain neutralization depends on a residue outside the epitope, which presumably modulates epitope accessibility on the virion. These studies highlight how the choice of virus strain can impact antibody responses and inform future epitope-based and germline-directed vaccine strategies against flavivirus infections.

## Materials and methods

Key resources can be found in Table S9.

**Table S9.** Key resources

Reagent or resource	Source	Identifier
**Antibodies**		
Monoclonal anti-ZIKV-E ZIKV-116	This paper	N/A
Monoclonal anti-WNV-E E60	Michael S. Diamond, WUSTL, (mdiamond@wustl.edu)	Oliphant et al., 2006
Monoclonal anti-WNV-E E53	Michael S. Diamond, WUSTL	Oliphant et al., 2006
Monoclonal anti-WNV WNV-E111	Michael S. Diamond, WUSTL	Oliphant et al., 2006
Monoclonal anti-DENV1 DENV1-E111	Michael S. Diamond, WUSTL	Austin et al., 2012
Monoclonal anti-DENV4 DENV4-E88	Michael S. Diamond, WUSTL	Sukupolvi-Petty et al., 2013
Monoclonal anti-JEV JEV-106	Michael S. Diamond, WUSTL	Fernandez et al., 2018
Monoclonal anti-POWV POWV-61	This paper, unpublished mAb	N/A
Goat anti-human IgG-HRP	Santa Cruz Biotechnology	Cat# sc-2907
Goat anti-mouse IgG-HRP	Santa Cruz Biotechnology	Cat# sc-2005
Goat anti-mouse-PE	BD Pharmingen	Cat# 550589
**Bacterial and virus strains**		
ZIKV strain: French Polynesia_H/PF/2013	Michael S. Diamond, WUSTL	Zhao et al., 2016
ZIKV strain: Uganda_MR-766	Michael S. Diamond, WUSTL	Zhao et al., 2016
ZIKV strain: Senegal_Dakar 41519	Michael S. Diamond, WUSTL	Zhao et al., 2016
DENV1 strain 16007	Michael S. Diamond, WUSTL	Shrestha et al., 2010
DENV1 strain West Pac-74	Michael S. Diamond, WUSTL	Shrestha et al., 2010
BL21-CodonPlus-RIL competent cells	Agilent Technologies	Cat# 230240
**Chemicals, peptides, and recombinant proteins**		
EZ-Link-NHS-PEG4-Biotin	Thermo Fisher	Cat# 21330
Recombinant ZIKV-DIII from H/PF/2013	This paper	GenBank: AHZ13508.1
Recombinant ZIKV-DIII from MR-766	This paper	GenBank: ANO46296.1
Recombinant DENV1-DIII from 16007	Austin et al., 2012	N/A
Recombinant DENV1-DIII from West Pac-74	Austin et al., 2012	N/A
Recombinant DENV2-DIII	Austin et al., 2012	N/A
Recombinant DENV3-DIII	Austin et al., 2012	N/A
Recombinant DENV4-DIII	Austin et al., 2012	N/A
Recombinant WNV-DIII from New York 1999	Nybakken et al., 2005	N/A
Recombinant WNV-E from New York 1999	Luca et al., 2012	N/A
Recombinant JEV-DIII from SA14-14-2	Fernandez et al., 2018	N/A
Recombinant SPOV-E from SM6 V-1	This paper	GenBank: YP_009227187.1
Recombinant POWV-DIII from Spooner	This paper	GenBank: ADK37756.1
**Critical commercial assays**		
RNEasy Mini Purification Kit	Qiagen	Cat# 74104
SuperScript III First-Strand Synthesis System Thermo Fisher for RT-PCR	Thermo Fisher	Cat# 18080-051
4–12% Bis-Tris NuPAGE gel system	Thermo Fisher	Cat# 18080-051
FuGENE 6 Transfection Reagent		Cat# E2691
**Deposited data**		
ZIKV-116 Fab in complex with DIII structure	This paper	PDB: 6PLK
**Experimental models: cell lines**		
Vero	ATCC	Cat# CCL-81
Human: FreeStyle 293F	Thermo Fisher	Cat# R79007
Human: FreeStyle 293F	Thermo Fisher	Cat# R79007
HEK 293T	ATCC	Cat# CRL-1573
Raji-DCSIGNR	Pierson laboratory	N/A
**Recombinant DNA**		
Plasmid: pML-huCG1	This paper	N/A
Plasmid: pET21a	This paper	N/A
**Software and algorithms**		
GraphPad Prism	GraphPad Software, Inc.	https://www.graphpad.com
FlowJo version 10	Tree Star	https://www.flowjo.com/solutions/flowjo/downloads
Biaevaluation 4.1	GE Healthcare	https://www.biacore.com/lifesciences/service/downloads/software_licenses/biaevaluation/
Phaser	McCoy et al., 2007	http://www.ccp4.ac.uk/html/phaser.html
Phenix	Adams et al., 2010	https://www.phenix-online.org/
Coot	Emsley et al., 2010	https://www2.mrc-lmb.cam.ac.uk/personal/pemsley/coot/
PDBePISA	Open source	www.ebi.ac.uk/pdbe/pisa/
PyMOL	Schrödinger	https://www.pymol.org/
Ligplot	McDonald and Thornton, 1994	https://sbgrid.org/software/titles/ligplot
Agilent QuikChange Primer Design program	Agilent Technologies	N/A

N/A, not applicable; WUSTL, Washington University in St. Louis.

### Viruses and cells

Vero, C6/36, and HEK 293T cells were maintained in DMEM supplemented with 7% FBS, 100 U/ml penicillin/streptomycin, 10 mM Hepes, 1 mM sodium pyruvate, and 1× nonessential amino acids. Raji cells (Raji-DCSIGNR) which express the attachment factor DC-SIGNR (dendritic cell–specific ICAM-3–grabbing nonintegrin related) were maintained in RPMI 1640 medium with the same media supplements.

### Virus and RVPs

ZIKV strains H/PF/2013 (French Polynesia, 2013) and MR-766 were grown in Vero cells at 37°C. DENV1 16007 (genotype 2) and West Pac-74 (genotype 4) were amplified in C6/36 *Aedes albopictus* cells at 28°C. ZIKV and DENV1 RVPs were generated as described previously (Dowd et al., 2016, 2015). HEK 293T cells were cotransfected with a plasmid encoding the structural C-prM-E proteins and another plasmid encoding a WNV replicon with a GFP reporter gene, which can be used to monitor infection by flow cytometry. RVP-containing supernatants were harvested between 72 and 120 h after transfection and frozen at −80°C.

### Focus-forming and reduction assays

A focus-forming assay (FFA) was used to determine virus titer on Vero cells. Vero cell monolayers were inoculated with virus or virus-mAb mixture and incubated at 37°C for 1 h. Cells then were overlaid with 1% (wt/vol) methylcellulose in MEM supplemented with 4% FBS and incubated at 37°C. 40 h (ZIKV) or 68 h (DENV) later, the cells were fixed with 4% paraformaldehyde and incubated with 500 ng/ml WNV-E60 mAb (Oliphant et al., 2006) in PBS buffer supplemented with 0.1% saponin and 0.02% Tween 20 for 2 h. After washing, cells were stained with HRP-conjugated goat anti-mouse IgG, and virus-infected foci were developed using TrueBlue peroxidase substrate (KPL) for 30 min and counted by an ImmunoSpot 5.0.37 macroanalyzer (Cellular Technologies).

Focus reduction neutralization tests (FRNT) were conducted with indicated ZIKV strains and mAbs in Vero cells in 96-well plates as described previously (Zhao et al., 2016). The infection frequency of the wells inoculated with virus in the presence of mAb was compared with that of wells inoculated with virus and medium alone. The IC_50_ values were determined using nonlinear regression analysis (GraphPad Prism).

### RVP titration and neutralization assay

ZIKV and DENV1 RVP neutralization assays were performed as previously described (Dowd et al., 2016, 2015). To determine virus titer, twofold dilutions of RVPs were used to infect Raji-DCSIGNR in duplicate technical replicates at 37°C. GFP-positive infected cells were detected by flow cytometry 40 h later. In subsequent neutralization assays, RVPs were sufficiently diluted to within the linear range of the virus-infectivity dose-response curve to ensure antibody excess at informative points. To measure neutralization, ZIKV or DENV1 RVPs were mixed with serial dilutions of mAb for 1 h at 37°C, followed by infection of Raji-DCSIGNR cells in duplicate technical replicates. Infections were performed at 37°C, and GFP-positive infected cells were quantified by flow cytometry 2 d later. Results were analyzed by nonlinear regression analysis to estimate the dilution of mAb required to inhibit 50% of infection (IC_50_).

### ELISA

All the recombinant E and DIII (5 µg/ml) from distinct flaviviruses were coated onto a MAXISORP 96-well plate (Nunc) and blocked with PBS supplemented with 0.02% Tween 20, 5% BSA, and 5% goat serum. 1 h later, plates were incubated with ZIKV-116 or control mAbs (WNV-E111 [Oliphant et al., 2006], DENV4-E88 [Sukupolvi-Petty et al., 2013], JEV-106 [Fernandez et al., 2018], and POWV-61 [unpublished mAb]), followed by HRP-conjugated anti-human or anti-mouse IgG (sc-2907 or sc-2005; Santa Cruz Biotechnology). The reaction was developed with 3,3′-5,5′ tetramethylbenzidine substrate followed by stopping with the addition of 1 N H_2_SO_4_, and the absorbance was measured at 450 nm.

### Protein production, purification, and crystallization

ZIKV DIII (H/PF/2013 and MR-766, residues 299–407), DENV1-4 DIII (residues 293–399; Austin et al., 2012), WNV DIII and E (New York, 1999; residues 296–401 [DIII] and residues 1–406 [E]; Luca et al., 2012; Nybakken et al., 2005), JEV DIII (SA14-14-2, residues 299–399; Fernandez et al., 2018), POWV DIII (Spooner, residues 298–399), ZIKV E (H/PF/2013, residues 1–407), and SPOV E (SM6 V-1, residues 1–411) were cloned into the pET21a vector (Novagen) and expressed as inclusion bodies in *Escherichia coli* BL21(DE3). Isolated inclusion bodies were solubilized and refolded as previously described (Dai et al., 2016; Nybakken et al., 2005). ZIKV-116 heavy and light chain variable regions were codon-optimized for mammalian expression and synthesized on a BioXP 3200 DNA synthesis instrument (SGI-DNA). The resulting gene fragments were cloned directly into CMV-driven mammalian expression vectors that had already been fused with C_H_1-2-3 for IgG1, C_H_1 for Fab, and C_K_ for IgK. The mAb or Fab heavy and light chain vectors were cotransfected into Expi293F or ExpiCHO cells (Thermo Fisher) for transient expression. After 7 d of culture, the culture supernatants were separated by centrifugation, filtered, and purified by fast protein liquid chromatography on an ÄKTA instrument using a HiTrap MabSelect Sure column (GE Healthcare) or a CaptureSelect IgG-CH1 column (Thermo Fisher), respectively. The purified mAb or Fab then was buffer-exchanged into PBS.

Purified ZIKV-116 Fab was incubated with excess DIII for 2 h followed by complex isolation through size exclusion chromatography (Superdex 75 16/600). The complexes were collected and crystallized in hanging drops at 20°C. Diffraction quality crystals were obtained in the condition of 30% 2-Methyl-2,4-pentanediol, 0.1 M imidazole, and 10% polyethylene glycol 4K, at a concentration of 12 mg/ml. Protein crystals were cryo-protected in reservoir solution with 15% ethylene glycol before being flash-cooled in liquid nitrogen.

### Structure determination and refinement

Diffraction data were collected at the Advanced Photon Source beamline 24-ID-C and processed with the HKL2000 program. Structural phase was determined by molecular replacement in Phenix (Adams et al., 2010) using the Phaser GUI (McCoy et al., 2007), and previously solved ZIKV DIII from the complex of ZV-67-DIII (PDB 5KVG) and human Fab structure from Z20-sE (PDB 5GZO) were used as the search models, respectively. Cycles of model building and refinement were performed in Coot (Emsley et al., 2010) and Phenix. The structural figures were generated in PyMOL software. The interaction of Fab and DIII was analyzed in the ligplot program suite using default settings (McDonald and Thornton, 1994). The buried surfaces and shape complementarity were calculated using PISA (http://www.ebi.ac.uk/msd-srv/prot_int/).

### BLI

The binding affinity of recombinant DIII with ZIKV-116 was monitored by BLI using an Octet-Red96 device (Pall ForteBio). ZIKV-116 and ZIKV-116-V.J.Rev mAbs were biotinylated using EZ-Link-NHS-PEG4-Biotin reagent and then loaded onto streptavidin biosensors (ForteBio). Binding at neutral pH was determined in HBS-EP buffer (10 mM Hepes, pH 7.4, 150 mM NaCl, 3 mM EDTA, 0.005% P20, and 3% BSA). Low pH binding was performed in a similar assay buffer with the pH adjusted to 5.5 using 2-ethanesulfonic acid buffer. Buffer alone with loaded mAb and sensor alone with the highest concentration of DIII were added in parallel to serve as negative controls. The experimental curves were analyzed using Biaevaluation 4.1 (GE Healthcare) and fitted by a 1:1 Langmuir fitting model to determine the associate constant (k_a_), dissociate constant (k_d_), kinetic binding affinity (K_D_, kinetic), and steady-state affinity (K_D_, equilibrium).

### Attachment inhibition assay

Attachment blockade was measured by qRT-PCR on an ABI 7500 Real Time-PCR system (Applied Biosystems). 2 × 10^5^ Vero cells were seeded in 24-well plates and incubated for 24 h. ZIKV (2 × 10^2^ focus-forming units [FFU]) were preincubated with ZIKV-116 or a human isotype control (anti-influenza IgG1) mAb (0.1 µg/ml, 1 µg/ml, 10 µg/ml) for 1 h, followed by addition of the mAb-virus complex to chilled Vero cells for 1 h at 4°C. Cells were extensively washed with cold DMEM on ice, and total RNA was extracted using an RNeasy Mini Kit (Qiagen). ZIKV RNA levels were determined using a Taqman RNA-to-Ct 1-Step Kit (Thermo Fisher) normalized to the internal control GAPDH. Primers and probes used are as follows: ZIKV-E-Fwd, 5′-CCA​CCA​ATG​TTC​TCT​TGC​AGA​CAT​ATT​G-3′; ZIKV-E-Rev, 5′-TTC​GGA​CAG​CCG​TTG​TCC​AAC​ACA​AG-3′; Probe ZIKV-E, 56-FAM/AGCCTACCT/ZEN/TGACAAGCAGTC/3IABkFQ; GAPDH-Fwd, 5′-TGT​AGT​TGA​GGT​CAA​TGA​AGG​G-3′; GAPDH-Rev, 5′-ACA​TCG​CTC​AGA​CAC​CAT​G-3′; Probe GAPDH, 56-FAM/AAGGTCGGA/ZEN/GTC​AAC​GGATTTGGTC/3IABkFQ.

### Pre- and post-attachment neutralization assays

For the pre-attachment inhibition assay, serial dilutions of ZIKV-116 were incubated with ZIKV (10^2^ FFU) for 1 h at 4°C followed by addition to chilled Vero cells for 1 h. The cells were washed three times with cold DMEM to remove unbound virus, then the plates were transferred to 37°C, and standard FFAs were performed as described above. For post-attachment inhibition assay, 10^2^ FFU of ZIKV was inoculated onto chilled Vero cells for 1 h at 4°C, and then cells were washed with cold media to remove unbound virus, followed by the addition of serial dilutions of ZIKV-116 for 1 h at 4°C. Finally, excess mAbs were washed away, and standard FFAs were conducted as described above.

### Plasma membrane fusion assay

Vero cells seeded in 96-well plates were rinsed once with PBS and then incubated with DMEM supplemented with 25 mM NH_4_Cl and 0.2% BSA (DMEM-NH_4_Cl) on ice for 15 min. ZIKV (multiplicity of infection of 80) was added to cells for 1 h at 4°C followed by three washes with chilled DMEM-NH_4_Cl to remove unbound virus. The cells were incubated with 100 µg/ml of mAbs or media without mAb for 1 h on ice. After washing with chilled DMEM-NH_4_Cl, cells were incubated with acidic medium (~pH 5.8) or neutral medium (pH 7.4) for 9 min at 37°C to induce viral fusion with cell membrane. Subsequently, cells were washed with DMEM-NH_4_Cl (pH 7.4) and incubated at 37°C with DMEM containing 25 mM NH_4_Cl to prevent endosomal-mediated viral fusion and secondary infection. The cells were fixed with 2% paraformaldehyde 24 h after infection and sequentially incubated with the cross-reactive WNV-E53 mAb (Oliphant et al., 2006; 0.5 µg/ml; recognize E protein) and goat anti-mouse PE-conjugated antibody (1:1,000). Finally, the cells were washed three times and subjected to flow cytometry analysis, and data were analyzed using FlowJo software.

### Statistical analysis

All functional results were analyzed using GraphPad Prism Software, and differences were evaluated by two-tailed *t* test or ANOVA test with post hoc corrections for multiple comparisons. A P value of <0.05 was considered to be significant.

### Data availability

The atomic coordinates of ZIKV-116 Fab in complex with ZIKV DIII have been submitted to the RCSB PDB with accession no. 6PLK.

### Online supplemental material

Fig. S1 shows that there are two ZIKV-116–DIII complexes in a crystallographic asymmetric unit, which is related to Fig. 2. Fig. S2 shows amino acid differences in DIII between two representative ZIKV strains (H/PF/2013 and MR-766) and two DENV1 strains (16007 and West Pac-74), which is related to Fig. 5. Fig. S3 shows time- and temperature-dependent neutralization curves for ZIKV-116 against ZIKV strain H/PF/2013. Fig. S4 shows the distance of residue 393 within structural ZIKV-116 epitope to residue 204^DENV1^/209^ZIKV^ from the same E protein or adjacent E protein on the virion surface. Table S1 presents crystallographic data collection and refinement statistics. Tables S2 and S3 provide van der Waals contacts for ZIKV-116–DIII complex. Tables S4–S6 present hydrogen bond contacts between ZIKV-116 and DIII. Table S7 shows the neutralizing values of ZIKV-116 and ZV-67 against different ZIKV strains. Table S8 provides binding parameters for ZIKV-116 to recombinant DIII protein of distinct ZIKV and DENV1 strains measured by BLI. Table S9 shows key resource information used in this study.
